# Assessment on urban lakes along the coastal region of Miri, NW Borneo: implication for hydrochemistry, water quality, and pollution risk

**DOI:** 10.1007/s11356-023-25172-9

**Published:** 2023-01-18

**Authors:** Inez Neysa anak Nyambar, Prasanna Mohan Viswanathan

**Affiliations:** Faculty of Engineering and Science, Department of Applied Sciences, Curtin University Malaysia, CDT 250, 98009 Miri, Sarawak Malaysia

**Keywords:** Lake water, Ion exchange, Irrigation, WQI, Pollution indices

## Abstract

The main purpose of this study is to assess the urban lake water quality along the coastal region of Miri City, Sarawak, East Malaysia. This study concentrates on the hydrochemical characteristics and the mechanisms controlling the suitability of the lakes for domestic, irrigation, and industrial purposes. A total of 15 lake water samples were collected and analysed for physical parameters, major ions, nutrients, BOD, COD, and heavy metals. The results show that Lakes 6, 7, and 8 commonly exceeded the National Drinking Water Quality Standards (NDWQS) limits, particularly in physical parameters and major ions. The cation dominance was Na^+^  > Mg^2+^  > K^+^  > Ca^2+^, while the anion dominance was Cl^−^  > HCO_3_^−^  > SO_4_^2−^. It is inferred that lake 8 was affected by the seawater intrusion through the inlet flow, resulting in an excess concentration of ions present in that lake. The concentrations of heavy metals in these lakes were considerably low, indicating that most of them were from the geogenic source. Most of the lakes were NaCl water type and the main controlling mechanism were weathering and ionic exchange processes, with anthropogenic impacts. Based on the calculated Water Quality Index (WQI) according to the National Water Quality Standards (NWQS), all lakes were suitable for irrigation use (class IV), while most of these lakes fell under the “polluted” index. Apart from that, based on the National Lake Water Quality Standards (NLWQS), all lakes were classified under category D, indicating that the lakes must be kept in good condition. According to the irrigation quality indices, lakes 1, 2, 3, 4, and 5 were the most suitable for irrigation purposes. As for metal pollution indices (HPI and HEI), lakes 1, 2, 3, 4, 5, and 15 were considered safe with low pollution status. From the multivariate statistical analysis, it is deduced that both anthropogenic and geogenic impacts mostly influenced the quality of these lake waters. The outcome of this study will help the policymakers under the national water department in sustainable management of water resources in this region.

**Supplementary Information:**

The online version contains supplementary material available at 10.1007/s11356-023-25172-9.

## Introduction

Water is an essential and basic necessity for all living things, especially to human beings; therefore, accessibility to clean water supply should be available at all times. While water is not only restricted to drinking and agricultural usages, it is also used for industrial, household, environmental, and recreational activities (Prasanth et al. 2012). Surface water is a form of natural water that is commonly the most accessible, with its sources including canals, lakes, ponds, rivers, and streams (Bwire et al. 2020). This water source faces serious environmental stress as it is the primary source of water for human needs; however, it threatened as a result of development activities (Seth et al. 2015). As nations around the world continue to develop, surface water quality has grown to be a major concern. The rapid rise in population growth, along with economic growth and the rate of urbanisation, is causing these resources to decline and induces high variances in water quality parameters (Aminiyan et al. 2016). The chemistry of water directly implies the water quality for several purposes; hence, monitoring and assessment of water qualities have attained considerable importance in recent times (Kumar 2013).

Before water is used for drinking, domestic, agricultural, or industrial purposes, it is necessary to test the water quality based on different physicochemical parameters due to water contents that might include bacteriological, dissolved, floating, microbiological, and suspended impurities (Bhateria and Jain 2016). The pollution of surface waters could be assessed in many ways, including biochemical oxygen demand (BOD) and chemical oxygen demand (COD). BOD is an essential indicator of water quality, where the pollution in the water contaminated by the disposal of industrial and domestic effluents is evaluated through BOD (Ravikumar et al. 2013; Sharifinia et al. 2013). The presence of biologically resistant organic substances and toxic conditions in the water is analysed through COD (Ma et al. 2020; Ravikumar et al. 2013). Heavy metals are considered to be significant contaminants in aquatic environments because of their toxicity, environmental persistence, and ability to enter food chains (Gupta et al. 2016).

Numerous physicochemical properties of soils are altered due to poor water quality, and various metabolic processes of plants are controlled by the unfavourable effects of such water (Yurtseven and Randhir 2020). Both physical and chemical effects of excessive amounts of dissolved ions would disturb and eventually damage the plant’s metabolism due to long-term use of poor-quality water (Ravikumar and Somashekar 2012). Therefore, the quality and suitability of irrigation water are determined through the total concentration of soluble salt measured by electrical conductivity (salinity hazard), the relative proportion of Na^+^ to other major cations, expressed as sodium adsorption ratio (sodium hazard), and the concentration of HCO_3_^−^ related to concentration of Mg^2+^  + Ca^2+^ (bicarbonate hazard).

Thivya et al. (2015) expressed that numerous hydrogeochemical studies that included the application of multivariate statistical analysis had been successful. This method acts as an analytical tool in reducing and organising massive hydrochemical datasets into groups sharing similar characteristics, and it serves as the fundamental purpose of interpreting relationships among variables applied for the classification of the original data set (Vasanthavigar et al. 2012). The methods include factor analysis (FA) and principal component analysis (PCA), which are helpful in the assessment of water quality and surface water management (Bilgin and Bayraktar 2021). Correlation analysis (CA) represents the degree of relationship between two random variables, where interpretation of the correlation would provide a plan of quick monitoring method on water quality (Ghazaryan and Chen 2016). Wang et al. (2015) stated that the relationship between observed parameters is indicated by FA through revealing multivariate patterns such as major factors that are used to simplify initial datasets. FA is generally used to narrow down and simplify outcomes obtained from PCA, where both methods are used to reduce data (Bilgin and Bayraktar 2021; Howladar et al. 2017). The rank of hydrogeochemical processes based on their order of importance and distinguishing the most dominant processes in the region could be achieved through FA (Thivya et al. 2013).

Natural or artificial lakes in Malaysia have been used for various purposes (Huang et al. 2015). Water Quality Index (WQI) and National Water Quality Standards for Malaysia (NWQS) are used in assessing the quality status of surface water (Maulud et al. 2021). WQI was introduced by the Department of Environment (DOE) and has been practiced in this country for approximately 25 years and has become the foundation in evaluating the environmental water quality, whereas the beneficial uses of watercourse according to WQI is classified by NWQS (Huang et al. 2015). Generally, NWQS acts as a guide in classifying the water quality status of surface water, along with determining the suitability for various uses and treatments necessary according to the WQI values (Maulud et al. 2021). The classification and quality standards of surface water are used for these lakes (Huang et al. 2015). The six parameters chosen for calculating standard WQI are suspended solids, pH, dissolved oxygen, COD, BOD, and ammoniacal nitrogen.

Previous studies were done by Prasanna et al. (2012a, b) on the selected lake water quality in Miri. In these studies, they mainly used metal concentration in the lakes to derive the metal-based water quality indices. Hence, the current study focuses on multiple hydrochemical data such as physical parameters, nutrients, major ions, and heavy metals to better understand the major geochemical processes that control the lake water quality in Miri. Since Miri is a developing city with its population growing with time, larger communities would clearly require more water supply. Assessing the water quality of lakes in Miri could act as a substitute for minor usages, such as other domestic uses besides drinking, small-scale agricultural activity, and other industries. These lake waters could also be treated to obtain a clean water supply whenever the main water supplies face an emergency. Recently, the Miri community had a water crisis due to the breakdown of water supply pipelines, maintained by LAKU Management. Therefore, the main objectives of this study is to assessing the surface water quality of lakes found within Miri, besides studying the hydrochemical properties of the lakes and the processes controlling them. Apart from that, this study aims to also identify the quality aspects of the lake waters to evaluate their suitability for domestic, irrigation, and industrial purposes. Lastly, the pollution condition of these lakes would be analysed according to the parameters and indices utilised in recent and relevant studies.

## Study area

The surface water in Miri City is easily accessible throughout the year, where the water is mainly used for domestic and irrigation purposes (Prasanna et al. 2012a). Besides being surrounded by the coastal region, Miri is also surrounded by agricultural, commercial, and industrial areas, along with squatted communities. Miri region is mostly composed of Neogene to Quaternary sedimentary rocks. The main lithology of the study area is alluvium, sandstones, siltstones, and mudstones, which belong to Miri, Tukau, Lambir and Sibuti Formations (Liechti et al. 1960; Adriansyah et al. 2016). Throughout the years, large-scale supplies of freshwater are being highly demanded as the human population and economic activities has grown in scale in the region. With the years passing by and the region evolving, such a situation could cause the quality and quantity of water supply to decline, due to poor management and water pollution of the existing resource. The study area experiences two monsoons which are the NE monsoon (November–March) and SW monsoon (May–September) with an inter-monsoon season (April and October). High rainfall was observed in the NE monsoon compared with the SW monsoon. In general, the Miri region experiences 3000 to 4000 mm of annual rainfall and a mean temperature of 26 °C, with 77% of relative humidity (Gan and Prasanna 2022).

**Fig. 1:**
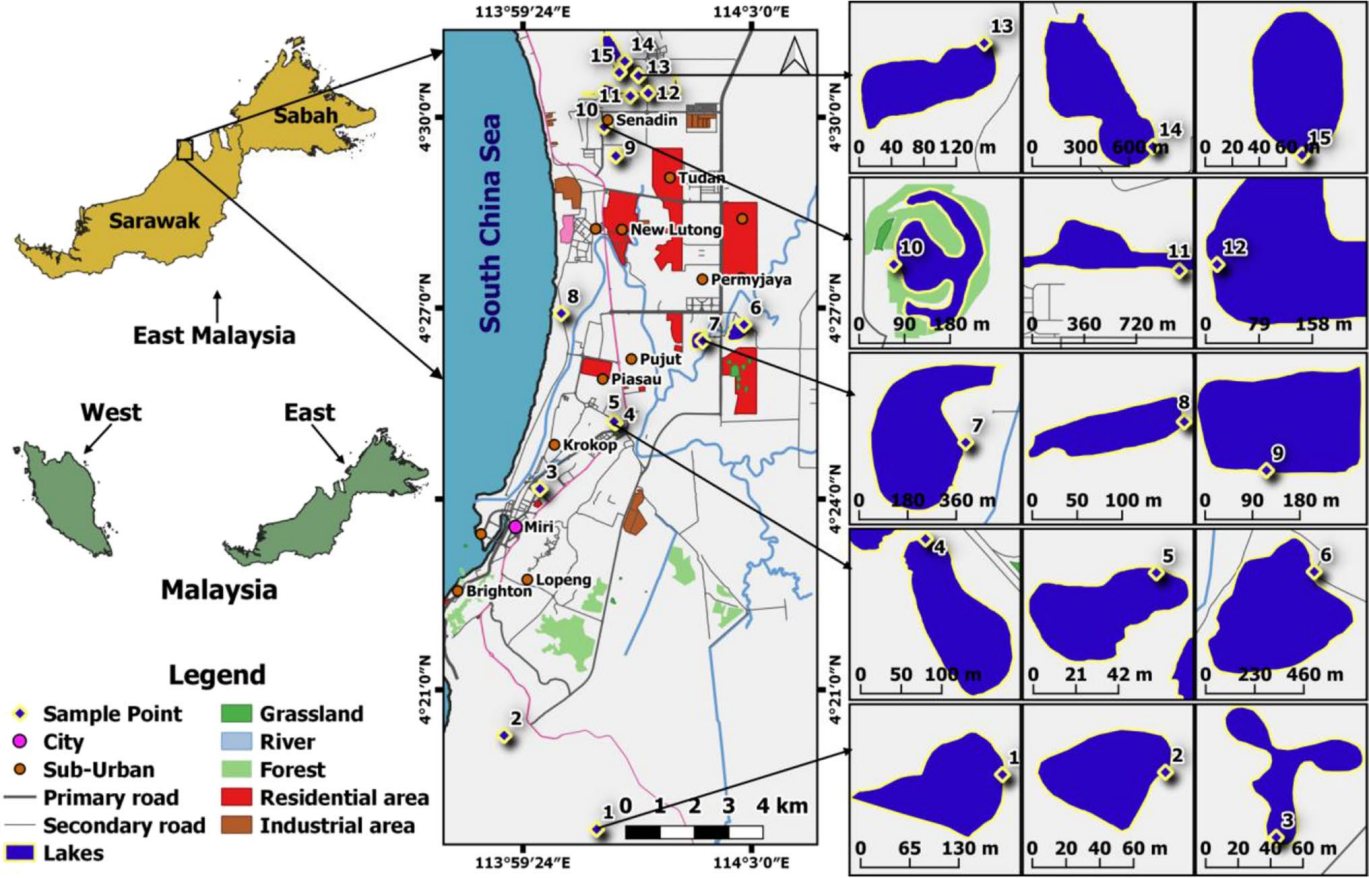
Map of the study area, along with individual dimensions of studied lakes

A total of 15 lakes were visited and studied for this research, with the area expanding from Senadin (North) to Kampung Lusut (South) of Miri. These lakes were initially scouted through Google Map, before proceeding to the scouted locations to check for their accessibility. The coordinates of these lakes are included in Supplementary 1, with brief descriptions of the nature of each lake. Aside from that, the study area map is presented in Fig. 1, where the dimension of each lake is mentioned respectively, and these lakes vary in size and shape. Lakes 2 and 3 have the smallest dimensions, roughly measured at 4800m^2^, while lake 14 has the biggest dimension, measured at 720,000m^2^. As for the land use pattern in this region, it differs depending on the locations of these lakes. Lakes 3, 4, and 5 are located at recreational parks that are frequently visited by local residents, whereas lakes 2, 9, 11, and 12 are located in residential areas, especially with both lakes 2 and 10 receiving domestic sewage discharges through the drainages.

## Methodology

### Sample collection and chemical analysis

All the lake water samples were collected on the same day (3 May 2021) using a water scooper from a depth of 5 to 10 cm from the water surface. Samples were collected from the bank of the lakes based on accessibility and safety reasons. The collected samples were transferred into 1L polyethylene bottles, and stored in a cool box (APHA 2005) for further analysis of nutrients (NO_3_^−^, NH_3_-N, PO_4_^3−^), BOD, COD, major ions (Cl^−^, HCO_3_^−^, SO_4_^2−^, Ca^2+^, Mg^2+^, Na^+^, K^+^), and heavy metals (Cu, Fe, Mn, Pb, Zn) in the laboratory. Before sampling, the bottles were rinsed with distilled water and the water to be sampled. Physicochemical parameters (pH, EC, DO, TDS, turbidity, temperature) were measured in situ using portable meters. The geographic coordinates of the lakes visited were obtained using a GPS. After collection, the bottles were kept in the lab’s fridge at 4 °C for further analysis. Before conducting lab analyses, the samples were filtered through 0.45-µm membrane filter papers using a filtration set. The nutrient analysis was done immediately on the following day of sampling, along with BOD and COD analyses. Nutrients were analysed in UV visible spectrophotometer (DR2800) using Hack test kits for NO_3_^−^ (cadmium reduction method), NH_3_-N (salicylate method), PO_4_^3−^ (ascorbic acid method), and SO_4_^2−^ (SulfaVer 4 method). COD method 8000 and BOD method 8043 from DR 2800 spectrophotometer procedures manual were used for COD and BOD analysis.

For COD, sample vials are placed in the preheated DRB200 reactor and with the protective lid of the reactor closed. The samples in the vials were then left to be heated for 2 h. After 2 h, the reactor was turned off and the vials were left for 20 min to be cooled off to 120 °C or less. The vials were also inverted several times while it is still hot. Then, the outside of the vial was cleansed thoroughly and inserted into the cell holder of the DR 2800 spectrophotometer, while lids were closed. Finally, the instrument read the barcode while selecting and performing the respective test. The results were recorded in mg/L COD.

Two sample volumes (150 ml and 300 ml) were chosen for this 5-day BOD test. The samples were prepared by stirring gently and adding sample volumes to the 300ml BOD bottles. Dilution water was prepared using a BOD nutrient buffer pillow. Three hundred-millilitre BOD bottle was filled with prepared dilution water which was used as a blank for the test. Lastly, a probe was used to measure each bottle’s dissolved oxygen concentration, including the blank. The prepared sample bottles were kept in an incubator at 20 °C for 5 days. After 5 days, the remaining dissolved oxygen in each of the prepared samples was measured. Initial and final readings of DO were measured to obtain the BOD value in mg/L.

Cl^−^, HCO_3_^−^, Ca^2+^, and Mg^2+^ were analysed using the titrimetric method (APHA 2005), while Na^+^ and K^+^ were analysed through flame atomic absorption spectrophotometer (AAS). The ionic balance error of total cations ( +) and total anions ( −) was calculated to establish the analytical precision for ionic measurements, as given below (Freeze and Cherry 1979).

1 $$\mathrm{Charge}\;\mathrm{balance}\;\mathrm{error}(\%)=\frac{\Sigma\;cation-\Sigma\;anions}{\Sigma\;cation+\Sigma\;anions}\times100$$

The acceptable error percentage was found to be between 5 and 10 (Domenico and Schwartz 1998).

For heavy metal analysis, the water samples were filtered and acidified to pH < 2 using nitric acid (30%). These samples were stored in a refrigerator at 4 °C until further processes such as digestion and heavy metal analysis using the flame atomic absorption spectrophotometer (AAS) (Perkin Elmer Analyst 400). For the digestion, 2 ml of concentrated HNO_3_ and 5 ml of concentrated HCl were added into 100 ml of water sample, where the beaker was then covered with a watch glass and left heated at 90 °C on a hot plate until the volume was reduced to 20 ml. First, calibration curves for the atomic absorption spectrometer’s accuracy were developed using standards made from stock and sub-stock. These reference curves were compiled prior to the analysis of water samples. The validity of the results was ensured by randomly comparing the values to the standards. The minimum absorbance values (characteristics concentration) of the metals in the instrument were Cu 0.15, Fe 3.6, Mn 0.016, Pb 0.18, and Zn 0.006 mg/L.

### Data analysis

#### Statistical analysis

As each sampling site was distinguished by a great number of physical and chemical variables, statistical analysis had allowed the lake water samples to be grouped where the correlations between the lake water samples and chemical parameters could be identified (Prasanna et al. 2010). Correlation analysis (CA) and factor analysis (FA) were used in this study, using Statistical Package of Social Studies (SPSS) software version 17.0. The variable inputs for FA were obtained from the data obtained from the laboratory analyses. Initial factor solutions were produced by the principal component analysis (PCA) method, which resulted in the preparation of a correlation matrix of the data. In order to compute the percentage of variation and cumulative percentage of the variance of the hydrogeochemical parameters of lake water, factors with eigenvalues greater than one were extracted (Thivya et al. 2013). Terminal factor solutions of orthogonal rotation for initial factors were determined using Kaiser’s varimax scheme (Prasanna et al. 2010). Factor scores of + 1 and greater indicate that the process has a great influence on the area, and factor scores of − 1 and lower indicate that the process does not influence the areas virtually, whereas near 0 scores indicate that the process has only moderate influence on the area (Thivya et al. 2015). These scores are normally extracted from factor loadings and scores represent the importance of a given factor in an area. Each sample’s factor scores were calculated by multiplying the factor score coefficient in a matrix with the standardised data; the result represents the significance of each factor score at each sample site (Thivya et al. 2015). The factor extraction was finalised with a minimum acceptable eigenvalue of 1, where the final factor was extracted using the varimax rotation method (Thivya et al. 2013). The original variables were transformed into new variables known as principal components (PCs), and these components were a combination of linearly related original variables, which was achieved through FA (Miyittah et al. 2020). This analysis particularly analyses the interrelationship among numerous variables in terms of their common fundamental dimensions known as factors, and this analysis also carries information.

#### Geochemical plots

The Piper plot (Fig. 7) included in this study was plotted using the Grapher by Golden Software. Gibbs plot, ionic ratio plots, USSL diagram, and Doneen plot were acquired through CHIDAM software (Chidambaram et al. 2021a).

### Calculation of water quality indices

#### Water Quality Index (WQI)

The formula and calculation for WQI provided by the National Water Quality Standards for Malaysia is as below:

2 $$WQI=\left(0.22*SIDO\right)+\left(0.19*SIBOD\right)+\left(0.16*SICOD\right)+\left(0.15*SIAN\right)+\left(0.16*SISS\right)+(0.12*SipH)$$

where the abbreviations are SIDO = SubIndex DO (%saturation), SIBOD = SubIndex BOD, SICOD = SubIndex COD, SIAN = SubIndex NH_3_-N, SISS = SubIndex SS, SipH = SubIndex pH, (0 ≤ WQI ≤ 100).

#### Irrigation indices

In this study, CHIDAM software (Chidambaram et al. 2021a) was used to calculate irrigation water quality indices such as sodium adsorption ratio (SAR), residual sodium carbonate (RSC), percentage sodium (%Na), Kelly’s ratio (KR), permeability index (PI), and magnesium hazard (MH) to evaluate the suitability of surface water for irrigation purposes. The equations of these indices are included in Supplementary 2.

#### Pollution indices

The equations for the pollution indices used in this study are presented in Supplementary 3. The overall quality of water with regard to metal is represented by the heavy metal pollution index (HPI) (Prasanna et al. 2012a). This index is useful in evaluating the impact of specific heavy metals on water quality and the insights into the suitability of surface water for human consumption (Gad et al. 2021). The index is created by assigning each chosen parameter a rating or weight (W_i_), where the rating/weight would reflect the overall water quality with respect to the recommended standard guidelines (S_i_) of each metal chosen as the parameter. The standard permitted value (S_i_) and the maximum desired value (I_i_) are the concentration limits for each parameter. Another index used in studying the condition of heavy metals in water is the heavy metal evaluation index (HEI). The conditions of water quality under metal stress are indicated by this index (Gad et al. 2021). The overall quality of water with regard to heavy metal is provided through both HPI and HEI indices (Prasanna et al. 2012b). The ratios of monitored values for the appropriate number of parameters and the maximum permissible concentrations for each parameter are used to calculate HEI, similar to HPI.

### Spatial maps

The sampled major lakes of the study area were digitised using the polygon tools of Google Earth Pro application and converted into polygon shape files in the QGIS. Google Earth Pro was used to digitise land use and land cover (LULC) in the study area, including water, grassland, forest, scrub, residential and industrial areas, and road networks. The QGIS platform created shape files from the digitised features. The basic map of the study area was created using appropriate symbols for each LULC class. GPS coordinates of each sample location of the selected lakes with the analytical results were loaded in an Excel spreadsheet and converted into a point shape file using the “Create point from table” tool of QGIS platform. The analysed results were then loaded into the attribute fields of respective polygon files of the sampled lakes using the “Join” tools with location point files. The study area map which showed all the sampled lakes was produced in the layout window of the QGIS application with all the map elements. Rule-based categorisation methods were applied to classify the lakes based on the analytical values of selected parameters. Using the QGIS layout tools, the classed layers were combined with the appropriate symbols to create categorised maps.

## Results

Results of the physicochemical parameters, major ions, nutrients, BOD, COD, and heavy metals are presented in Tables 1 and 2. These results were compared with the acceptable value of recommended raw water quality by the National Drinking Water Quality Standard (NDWQS 2016) and the WHO standard values (Table 3).

**Table 1:** Physicochemical parameters and concentrations of major ions present in the 15 lake water samples

Lake number	Location name	Parameters											
		pH	EC (µS/cm)	TDS (mg/L)	DO (mg/L)	Turbidity (NTU)	Temp (°C)	Cl− (mg/L)	HCO_3_− (mg/L)	Ca^2+^ (mg/L)	Mg^2+^ (mg/L)	Na^+^ (mg/L)	K^+^ (mg/L)
1	Kpg. Lusut	7.73	27.9	13.94	5.5	2.02	32.6	106.35	61	6	1.2	1.148	0.324
2	Jln. Airport	8.84	349	171.9	14.7	18.4	33.7	141.8	122	20	6	29.68	8.72
3	Jln. Kipas	6.93	223	114.3	4.9	21.3	32.2	127.62	167.7	24	3.6	2.912	6.22
4	Tmn Bulatan Lake (a)	8.84	120.8	60.9	8.1	29.8	33.8	99.26	97.6	16	1.2	2.128	1.17
5	Tmn Bulatan Lake (b)	8.01	101.5	50.9	5.6	3.16	32.8	113.44	67.1	10	2.4	1.464	0.187
6	Go-kart Lake	6.6	2470	1229.7	3.8	3.39	30.8	751.5	67.1	18	34.8	357	21.376
7	South Lake	6.97	2200	1077	6.2	3.11	32.2	595.56	85.4	12	25.2	332	15.752
8	Jln. Pantai	9.28	16,900	8490	4.2	19	33.7	4098	109.8	70	200.4	2760	124.672
9	Jln. Maigold	8.15	418.5	210	4.7	32.6	32.6	155.98	85.4	12	4.8	51.6	4.964
10	Kolam MGP	8.21	478.5	241	5.9	36.2	33.1	191.43	61	10	4.8	64.2	5.92
11	Jln. Curtin (a)	6.45	1357	678	4.4	0.64	31.4	411.22	79.3	14	14.4	158.4	7.94
12	Jln. Curtin (b)	6.72	235	118	2.6	1.59	32.9	113.44	48.8	10	6	29.84	1.423
13	Within Curtin	6.79	661	331	3.2	15.1	34.1	177.25	170.8	16	6	81.8	8.38
14	Curtin main lake	6.65	326	163.2	3.9	2.49	35.6	141.8	42.7	8	3.6	43.4	1.513
15	Curtin Lakeside	6.82	794	398	5.4	8.99	34.2	241.06	61	12	8.4	128.8	6.152

**Table 2:** Nutrients, BOD, COD, and concentrations of heavy metals present in the 15 lake water samples

		Parameters										
Lake number	Location name	NO_3_−N (mg/L)	NH_3_−N (mg/L)	PO_4_^−^ (mg/L)	SO_4_^2^−(mg/L)	BOD (mg/L)	COD (mg/L)	Cu (mg/L)	Fe (mg/L)	Mn (mg/L)	Pb (mg/L)	Zn (mg/L)
1	Kpg. Lusut	0.01	0.2	0.95	0.3	0.39	32	0.019	0.533	0.011	0.035	0.016
2	Jln. Airport	0.04	3.7	0.97	42	8.61	53	0.042	0.182	0.077	0.01	0.03
3	Jln. Kipas	0.01	0.21	0.87	0.1	8.46	34	0.071	0.127	0.016	0.015	0.027
4	Tmn Bulatan Lake (a)	0.02	0.2	1.36	0.1	9.52	70	0.029	0.159	0.009	0.025	0.038
5	Tmn Bulatan Lake (b)	0.03	0.13	0.95	3	0.35	111	0.019	0.431	0.011	0.005	0.02
6	Go-kart Lake	0.01	0.11	1.18	90	1.37	112	0.029	0.695	0.019	0.061	0.3
7	South Lake	0.03	0.13	1.86	36	1.9	67	0.022	0.064	0.014	0.055	0.028
8	Jln. Pantai	0.01	0.09	0.44	480	3.68	118	0.062	0.563	0.03	0.166	0.045
9	Jln. Maigold	0.01	0.17	0.78	29	7.08	52	0.024	0.124	0.024	0.049	0.035
10	Kolam MGP	0.02	0.01	0.94	0.1	10.36	57	0.013	0.596	0.031	0.044	0.08
11	Jln. Curtin (a)	0.01	0.04	1.14	12	0.55	113	0.019	0.55	0.015	0.051	0.025
12	Jln. Curtin (b)	0.03	0.1	0.95	0.3	0.33	128	0.014	1.222	0.019	0.033	0.019
13	Within Curtin	0.02	0.01	0.83	0.1	3.54	83	0.041	3.38	0.029	0.029	0.014
14	Curtin main lake	0.01	0.24	0.96	0.2	0.12	86	0.006	1.46	0.026	0.02	0.02
15	Curtin Lakeside	0.01	0.03	1.34	0.2	0.19	83	0.015	1.093	0.019	0.03	0.023

**Table 3:** Maximum, minimum, and average values of parameters were recorded, along with Malaysia’s Drinking Water Quality Standard (NDWQS)’s acceptable values for recommended raw water quality, WHO guideline values, and the number of samples exceeding the limits

Parameters	Maximum	Minimum	Average	NDWQS (acceptable value)	WHO (guideline value)	Sample exceeding limit	
						NDWQS	WHO
pH	9.28	6.45	7.53	5.5–9.0	6.5–8.5	1	3
EC, µS/cm	16,900	27.9	1777.48	1000	400	4	8
TDS, mg/L	8490.00	13.94	889.86	1500	500	1	4
DO, mg/L	14.7	2.6	5.54	-	5	-	7
Turbidity, NTU	36.2	0.64	13.19	1000	5	-	8
Temp, °C	35.6	30.8	33.1	-	30	-	15
Cl^−^, mg/L	4098.00	99.26	497.71	250	200	4	5
HCO_3_^−^, mg/L	170.8	42.7	88.45	-	500	-	-
SO_4_^2−^, mg/L	480	0.1	46.2	250	250	1	1
Ca^2+^, mg/L	70.00	6	17.2	-	75	-	-
Mg^2+^, mg/L	200.4	1.2	21.5	150	30	1	2
Na^+^, mg/L	2760.00	1.148	269.625	200	200	3	3
K^+^, mg/L	124.67	0.187	14.314	-	12	-	3
NO_3_-N, mg/L	0.04	0.01	0.02	10	45	-	-
NH_3_-N, mg/L	3.7	0.01	0.36	-	-	-	-
PO_4_^−^, mg/L	1.86	0.44	1.03	-	5	-	-
BOD, mg/L	10.36	0.12	3.76	6	5	5	5
COD, mg/L	128	32	79.9	10	-	15	-
Cu, mg/L	0.071	0.006	0.028333	1	2	-	-
Fe, mg/L	3.38	0.064	0.745267	1.0	0.3	4	10
Mn, mg/L	0.077	0.009	0.023333	0.2	0.4	-	-
Pb, mg/L	0.166	0.005	0.041867	0.05	0.01	3	14
Zn, mg/L	0.3	0.014	0.048	3	5	-	-

### Physicochemical parameters

**Fig. 2:**
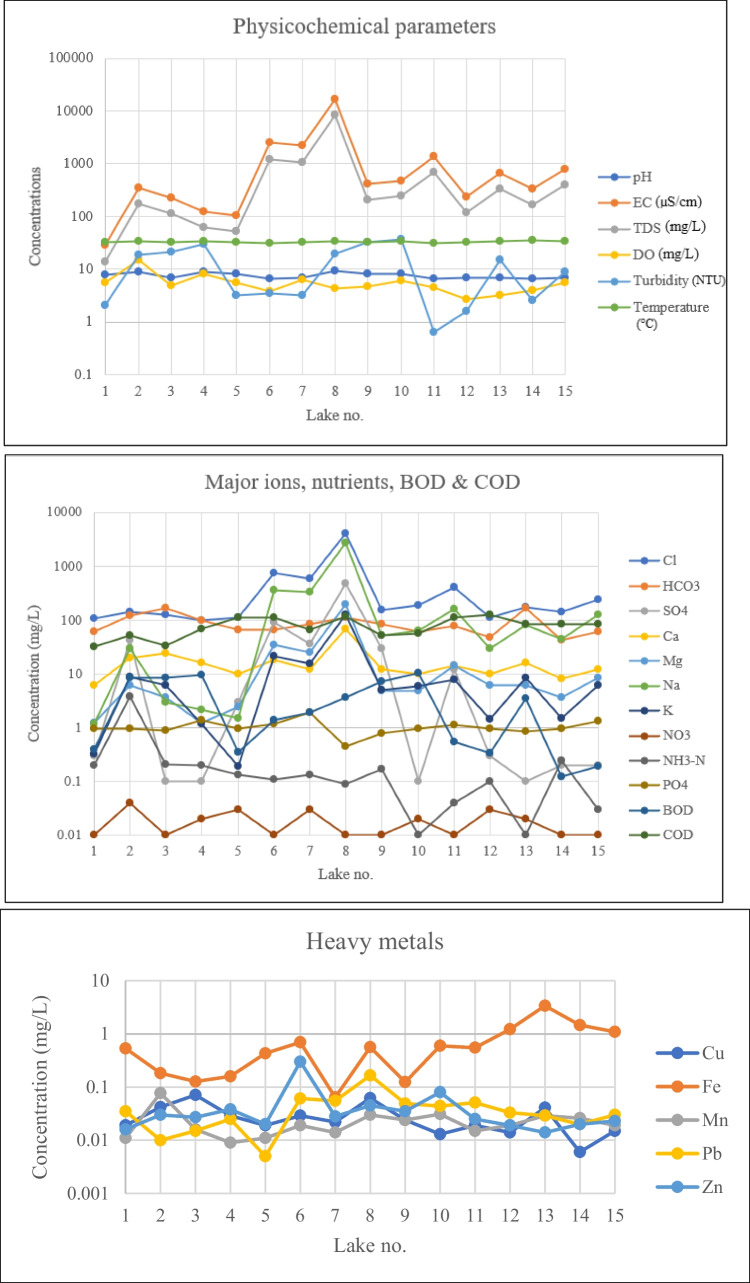
The concentration of physicochemical parameters, nutrients, BOD, COD, major ions, and heavy metals in the studied lakes

The pH values ranged from 6.45 to 9.28, where lake 11 had the lowest pH and lake 8 had the highest pH. It showed that these water samples are acidic to alkaline, where 50% of the samples are acidic in nature. Only lake 8 exceeded the NDWQS limit (9), while the other lakes were all within limit. EC values ranged from 27.9 to 16,900 µS/cm, in which lake 1 had the lowest value and lake 8 had the highest value (Fig. 2). Four lakes (lakes 6, 7, 8, 11) exceeded the NDWQS limit (1000 µS/cm), while the other lakes were within the limit. The measured TDS values ranged from 13.94 to 8490 mg/L. Lake 1 had the lowest TDS value, while lake 8 had the highest TDS value. Only lake 8 exceeded the NDWQS limit, while the other lakes had values below the limit. DO values ranged from 2.6 to 14.7 mg/L, where lake 2 had the highest value, and lake 12 had the lowest value. No limit was stated for NDWQS; hence, the DO standard limit provided by Malaysia’s NWQS (2006) was considered. The highest standard limit of the 5 classes is 7 mg/L, resulting in lakes 2 and 4 as the only lakes exceeding the limit, while the other lakes had DO values below the limit. Turbidity ranged from 0.64 to 36.2 NTU, with lake 11 having the lowest value and lake 10 with the highest value, making all 15 lakes within the NDWQS limit (1000 NTU). The temperature recorded ranged from 30.8 to 35.6 °C, where lake 6 had the lowest temperature and lake 14 had the highest temperature. No limit was designated for NDWQS; hence, the WHO (2011) limit (30 °C) was considered, and based on it, all 15 locations exceeded the limit.

### Major ions

Cl^−^ concentrations ranged from 99.26 to 4098 mg/L, with the lowest concentration at lake 4, while the highest concentration at lake 8. Four lakes (lakes 6, 7, 8, 11) exceeded the limit, while the other 11 lakes were below the limit (Fig. 2). For HCO_3_^−^, the samples ranged from 42.7 to 170.8 mg/L, with the highest concentration at lake 13, and the lowest concentration at lake 14. No value was stated for NDWQS; therefore, the WHO (2011) limit was used instead, making all the lakes below the limit. CO_3_^2−^ was only present in lake 8, with a concentration of 24 mg/L. The Ca^2+^ concentrations ranged from 6 to 70 mg/L, with lake 1 having the lowest concentration and lake 8 with the highest concentration. Based on the WHO (2011), all samples were below the limit, as there was no limit stated for NDWQS. Mg^2+^ concentrations ranged from 1.2 to 200.4 mg/L, with the lowest concentration at lake 1 and 4, while lake 8 had the highest concentration, and only lake 8 exceeded the limit. Na^+^ concentrations ranged from 1.15 to 2760 mg/L, with lake 1 having the lowest concentration and lake 8 having the highest concentration. Twelve lakes had concentrations within the limit, whereas lakes 6, 7, and 8 exceeded the limit. K^+^ concentrations ranged from 0.19 to 124.67 mg/L, with the highest concentration at lake 8, and the lowest concentration at lake 5. No limit was stated for NDWQS; hence, the WHO (2011) limit was used instead, making 12 lakes having concentrations below the limit, and lakes 6, 7, and 8 exceeded the limit. SO_4_^2−^ concentrations ranged from 0.1 to 480 mg/L, with the highest concentration at lake 8 and the lowest concentration at lakes 3, 4, 10, and 13. Only lake 8 exceeded the limit, whereas the other lakes were below the limit.

### Nutrients, BOD, and COD

Nitrate (NO_3_^−^) concentrations ranged from 0.01 to 0.04 mg/L, with the highest concentration at lake 2, while the lowest concentration at lakes 1, 3, 6, 8, 9, 11, 14, and 15, and all the lakes were below the limit. Nitrogen ammonia (NH_3_-N) concentrations ranged from 0.01 to 3.7 mg/L, with the lowest concentration at lakes 13 and 15, while the highest concentration at lake 2 (Fig. 2). No limit was proposed for NDWQS; hence, the NWQS (2006) limit was considered. Concentration values above 2.7 mg/L are classified as class V, and based on this, only lake 2 was in this class, while the other lakes had concentrations much lower than 2.7 mg/L. Phosphate (PO_4_^3−^) concentrations ranged from 0.44 to 1.86 mg/L, with the lowest concentration at lake 8, while the highest concentration value at lake 7. No value was appointed by NDWQS; hence, the WHO (2011) value was used instead, where all the lakes were below the limit. BOD values ranged from 0.12 to 10.36 mg/L, with lake 14 having the lowest value and the highest value at lake 10. Five lakes (lakes 2, 3, 4, 9, 10) had values exceeding the limit, while the other lakes were within the limit. COD values ranged from 32 to 128 mg/L, with the lowest reading at lake 1 and the highest reading at lake 12, making all the lakes exceeding the limit.

### Heavy metals

Cu concentrations ranged from 0.006 to 0.071 mg/L, with lake 14 having the lowest value, and the highest value at lake 3, and all the lakes were below the limit. Fe concentrations ranged from 0.064 to 3.38 mg/L, where the highest concentration was at lake 13, and the lowest concentration was at lake 7 (Fig. 2). Lakes 12, 13, 14, and 15 exceeded the limit, while the other lakes were below the limit. Mn concentrations ranged from 0.009 to 0.077 mg/L, with the lowest value at lake 4 and the highest value at lake 2, making all the lakes below the limit. Pb concentrations ranged from 0.005 to 0.166 mg/L, with the highest value at lake 8, and the lowest value at lake 5. The lakes exceeding the limit were lakes 6, 7, and 11, whereas the other lakes were below the limit. Zn concentrations ranged from 0.014 to 0.3 mg/L, with the lowest reading at lake 13, while the highest reading at lake 6, and by this, all the lakes were below the limit.

### Water quality indices

#### Irrigation water indices

Concentrations of the major ions obtained were used in calculating the irrigation water quality indices mentioned in the methodology. For SAR, 12 samples were “excellent” (0–10) for irrigation, while 2 samples were “good” (10–18), and a sample was “poor” (> 26). Based on Wilcox (1955) Na% classification, 4 samples were “excellent” (0–20%) for irrigation, whereas 2 samples were “permissible” (40–60%). Meanwhile, 5 samples were “doubtful” (60–80%), and 4 samples were “unsuitable” (> 80%). According to Eaton’s (1950) classification for Na%, 6 samples were “safe” for irrigation, while the other 9 samples were “unsafe”. Thirteen samples had “good” (< 1.25) RSC values for irrigation. Out of these 13 samples, 6 samples had negative values, indicating that these samples have both their Ca^2+^ and Mg^2+^ ions in the water that were being neutralised by excess amounts of Na^+^ ions present in the water, resulting in them being precipitated as CO_2_ instead (Dash and Kalamdhad 2021). The other 2 samples had “medium” (1.25–2.5) RSC values. For PI, all the samples were classified as class I (> 75%), making the waters good and suitable for irrigation use. As for KR, 5 samples were “safe” (KR < 1) for irrigation use, while the 10 other samples were “unsafe” (KR > 1). For MH, 10 samples were “safe” (MH < 50), whereas the other 5 lakes were “unsafe” (MH > 50). Therefore, the calculated irrigation water quality indices are presented in Supplementary 4, while the classifications of lakes according to distinct classes of the indices are presented in Supplementary 5.

#### Heavy metal pollution indices

**Fig. 3:**
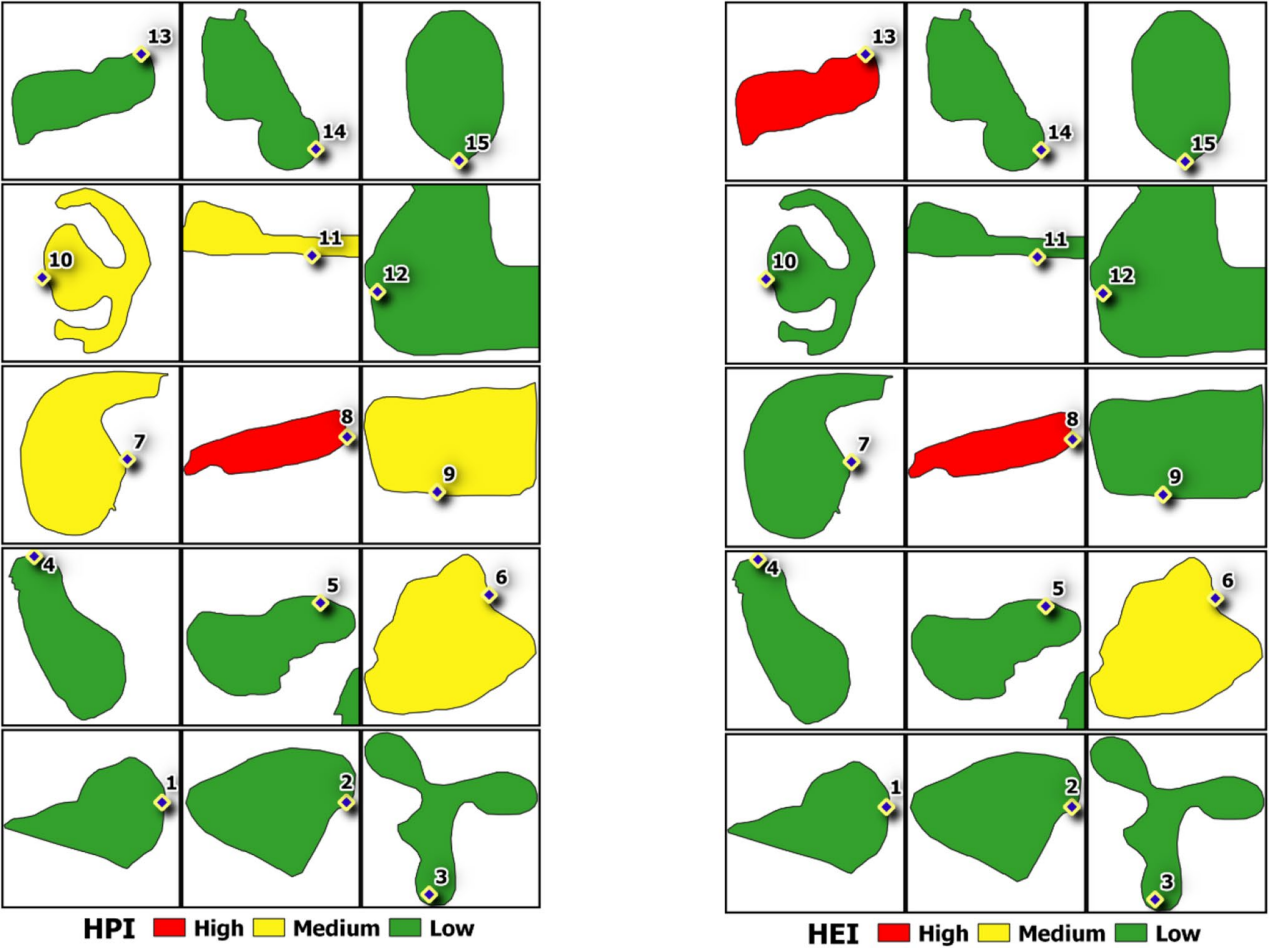
Spatial maps show the classification of lakes based on their HPI and HEI values, respectively

Concentrations of heavy metals obtained were used in both HPI and HEI equations, and the outcomes are presented in Supplementary 6. For HPI, the values ranged from 3.6 to 284.8, with the lowest value at lake 2 and the highest value at lake 8. These HPI values were subdivided into 3 categories based on the degree of pollution using mean values, which are “low” (< 60), “medium” (60–120), and “high” (> 120). Referring to Fig. 3, 9 lakes are classified as “low”, 5 lakes as “medium”, and only lake 8 as “high”. Next, the HEI values ranged from 0.6 to 4.2. Lakes 3 and 5 shared the lowest value, while lake 13 had the highest value. Similar to HPI, HEI is also subdivided into 3 categories based on the different degrees of pollution using mean values, namely “low” (< 2), “medium” (2–4), and “high” (> 4). Based on this, and referring to Fig. 3, 10 lakes have “low” HEI values, while 3 lakes have “medium” values, and 2 lakes with “high” values.

### Statistical analysis

Through correlation analysis (CA), the studied parameters were compared with each other to observe their interrelationship, where the threshold correlation value of 0.5 was considered. It was discerned that pH showed a good correlation with DO, turbidity, Ca^2+^, SO_4_^2−^, and BOD (Supplementary 7). EC showed a good correlation with PO_4_^3−^ and Zn, while TDS had a good correlation with Cl^−^, Ca^2+^, Mg^2+^, Na^+^, K^+^, SO_4_^2+^, Cu, and Pb. DO showed a good correlation with NO_3_^−^, NH_3_-N, BOD, and Mn. Turbidity showed a good correlation with temperature and BOD, while temperature only had a good correlation with BOD. Cl^−^ showed a good correlation with Ca^2+^, Mg^2+^, Na^+^, K^+^, SO_4_^2−^, Cu, and Pb, while HCO_3_^−^ showed a good correlation with BOD and Cu. Ca^2+^ had good correlation with Mg^2+^, Na^+^, K^+^, Cu, and Pb, while Mg^2+^ showed good correlation with Na^+^, K^+^, SO_4_^2−^, and Pb. Na^+^ showed a good correlation with K^+^, SO_4_^2+^, Cu, and Pb, while K^+^ showed a good correlation with SO_4_^2−^, Cu, and Pb. NO_3_^−^ showed a good correlation with NH_3_-N and Mn, while NH_3_-N only had a good correlation with Mn. However, PO_4_^3−^ did not show any correlations with any other parameters. SO_4_^2−^ showed a good correlation with Cu and Pb, and no correlations were observed between BOD and COD and heavy metals. There were no correlations between heavy metals.

From factor analysis (FA), 6 factors were achieved, and their % of variance ranged from 37.45 to 7.18% (Table 4). The 1^st^ factor has the highest %, and the parameters falling under this factor were TDS, Cl^−^, Ca^2+^, Mg^2+^, Na^+^, K^+^, SO_4_^2−^, and Pb. The 2^nd^ factor with 14.29% had DO, NO_3_^−^, NH_3_-N, and Mn, while the 3^rd^ factor with 12.35% had pH, turbidity, temperature, and BOD. The 4^th^ factor with 10.30% had HCO_3_^−^ and Cu, whereas the 5^th^ factor with 8.91% had EC, PO_4_^3−^, and Zn. Lastly, the 6^th^ factor with the lowest % only had Fe.

**Table 4:** Factor analysis

Rotated component matrix						
	Component					
	1	2	3	4	5	6
pH	0.463636	0.398851	0.48596	0.008255	− 0.39423	− 0.34733
EC	− 0.0612	− 0.0697	− 0.25909	− 0.03691	0.924165	− 0.09407
TDS	0.992834	− 0.0511	− 0.01972	0.051464	− 0.01678	0.011183
DO	− 0.11919	0.842453	0.178235	0.140511	− 0.06759	− 0.41205
Turbidity	0.074484	0.05474	0.881308	0.281173	− 0.15906	− 0.12054
Temp	− 0.07435	− 0.01265	0.818263	− 0.35274	0.112954	0.124542
Cl^−^	0.99407	− 0.05549	− 0.02217	0.044466	0.003606	0.000802
HCO_3_^−^	0.081364	0.134789	0.095242	0.933911	− 0.06737	0.162626
Ca^2+^	0.940015	0.045353	0.051447	0.302197	− 0.06783	0.01326
Mg^2+^	0.995194	− 0.04245	− 0.02626	0.047693	0.00022	0.009894
Na^+^	0.992289	− 0.05135	− 0.0173	0.043327	− 0.02921	0.009887
K^+^	0.991211	− 0.01552	0.009895	0.097217	0.00115	0.023244
NO_3_^−^N	− 0.20042	0.768713	− 0.08504	− 0.12858	− 0.06114	− 0.03834
NH_3_-N	− 0.05794	0.943242	0.02616	0.18276	− 0.04954	− 0.09683
PO_4_^−^	− 0.43728	0.010332	− 0.29309	− 0.14741	0.468838	− 0.49485
SO_4_^2−^	0.995646	0.022792	0.007594	0.066358	− 0.00786	− 0.00407
BOD	− 0.04458	0.265671	0.80968	0.407884	− 0.06403	− 0.21697
COD	0.417846	− 0.05829	− 0.42673	− 0.42614	0.190391	0.391195
Cu	0.46371	0.0902	0.087539	0.839027	− 0.05086	− 0.0234
Fe	− 0.11488	− 0.11936	− 0.16187	0.088016	− 0.03649	0.885881
Mn	0.126448	0.871408	0.232754	0.143042	− 0.00172	0.22937
Zn	0.154979	− 0.06309	0.151328	− 0.07216	0.835546	0.051845
Pb	0.937354	− 0.19805	0.058892	− 0.04175	0.132133	− 0.01887

Extraction method: principal component analysis

Rotation method: varimax with Kaiser normalisation

## Discussion

### Hydrochemistry

The cation dominance order for these lakes was Na^+^  > Mg^2+^  > K^+^  > Ca^2+^, while the anion dominance order was Cl^−^  > HCO_3_^−^  > SO_4_^2−^. Na^+^ and Cl^−^ are the most dominant ions, which are contributed by natural occurrences, such as seawater intrusion and ionic exchange. Excessively high concentrations of these two ions were recorded at lake 8, where this lake is located near Lutong Beach, and it was observed that the lake has a small inlet of seawater flowing into the lake. The highest pH was also recorded at this lake, which is caused by the intrusion of saltwater, and according to Singaraja et al. (2013), higher EC suggests the enrichment of salts in surface water. Besides seawater effect, excessively high Na^+^ concentration in the lakes, especially lakes 6 and 7, is possibly due to ionic exchange process (Chidambaram et al. 2010; Singaraja et al. 2013). Lakes 6 and 7 are assumed to receive water flow from the river nearby, Sungai Adong, as shown in Fig. 1; hence, the ionic exchange process could occur more in these lakes compared to the other lakes that are more isolated from other water bodies. The huge difference between the concentrations of Mg^2+^ and Ca^2+^ observed in lake 8, where Mg^2+^ is greater than Ca^2+^, is caused by the effect of seawater (Mondal et al. 2008; Singaraja et al. 2013). High Cl^−^ concentration at lake 8 is caused by the intrusion of seawater and it was also stated that the intrusion is accountable for the high SO_4_^2−^ concentration. High Cl^−^ concentration is also contributed by the inflow of wastewater or sewage (Shamsuddin et al. 2019). Ion exchange could potentially take place upon contact between freshwater systems with saline water. The high values of Na^+^, Ca^2+^, Mg^2+^, and K^+^ could be due to cationic contribution from the seawater and or alkali feldspar experiencing weathering (Prasanna et al. 2009). Signs of rock-water interaction and natural water recharge are exhibited through the presence of Ca^2+^, Mg^2+^, and HCO_3_^−^. Similar to Cl^−^, the Na^+^ concentration is contributed by other natural sources such as soil/rock-water interactions, and atmospheric deposition (Nosrati 2015; Sivakarun et al. 2020). High Mg^2+^ concentrations in waters are caused by Mg^2+^ ions being washed away from rock and later ending up in the waters (Mallick 2017). Excessive values of Na^+^ and K^+^ present in the waters are also linked to mineral dissolution, in addition to their abundance in nature and high solubility in water (Khadija et al. 2021). Hence, this could be considered a reason for the concentrations of these two ions measured in the 15 lakes studied. pH readings for some of the studied lakes indicate acidic nature, which could enhance the dissolution of minerals during weathering processes (Paul et al. 2019).

The analysis of NH_3_-N could be used to distinguish pollution caused by domestic sewage, animal faeces, and crop fertilisers used for agriculture (Maulud et al. 2021). Based on the highest NH_3_-N value recorded at lake 2, it is contributed by anthropogenic sources, as the lake receives domestic sewage from the drainage of the neighbourhood located opposite the lake. High BOD concentrations recorded in these lakes were mostly from domestic sewage. Dead plants were also found floating on the water surface, and according to Al-Badaii et al. (2013), the decaying process of natural plants could cause these high concentrations. The concentration of DO present in any aquatic ecosystem is directly controlled by BOD (Kumari and Sharma 2019). High concentrations of BOD would also cause the concentration of DO to decrease due to the consumption of DO by the bacteria (Maulud et al. 2021). In general, the COD level is directly proportional to the level of water pollution (Maulud et al. 2021). Sewage discharge, such as domestic sewage and food outlet sewage, along with broad usage of both organic and chemical fertilisers from agricultural activities, contributes to high COD concentration (Al-Badaii et al. 2013; Maulud et al. 2021). The studied lakes with high COD concentrations are assumed to be caused by sewage discharge and the usage of fertilisers. Higher NO_3_^−^ concentration in water would produce a great oxygen demand and cause a massive quantity of algal growth (Maulud et al. 2021). Similar to NH_3_-N, the highest NO_3_^−^ was recorded at lake 2, where these two parameters showed a good correlation in CA, indicating that the sources contributing to NO_3_^−^ could also be from sewage discharge. EC and TDS are suggested to be directly proportional to each other, as EC values are normally affected by the quantity of dissolved solids present in the waters (Meride and Ayenew 2016). Based on the considerably low PO_4_^3−^ concentrations for all the lakes, it could be from natural sources such as weathering of PO_4_^3−^-rich minerals present in the basin (Ma et al. 2020).

### Classification of lakes

The 15 lakes were classified into 4 categories (categories A, B, C, and D), as defined by NLWQS (2015). In all 15 lakes, temperature and total phosphorus exceeded the limits of all 4 categories. As shown in Supplementary 8, all 15 lakes are classified in category D, which meant that these lakes are managed for minimal preservation of good aquatic life in the lakes; in addition, the lakes in this category must be maintained in good condition. Besides that, 10 lakes are classified under category C, where these lakes need to be managed for preserving aquatic life and biodiversity reasons. Seven lakes are classified in category B, indicating that these lakes are managed for secondary body contact recreation purposes. Lastly, 3 lakes are classified under category A, which meant that these lakes are managed for primary body contact recreation, where swimming is included in the recreation purpose.

**Fig. 4:**
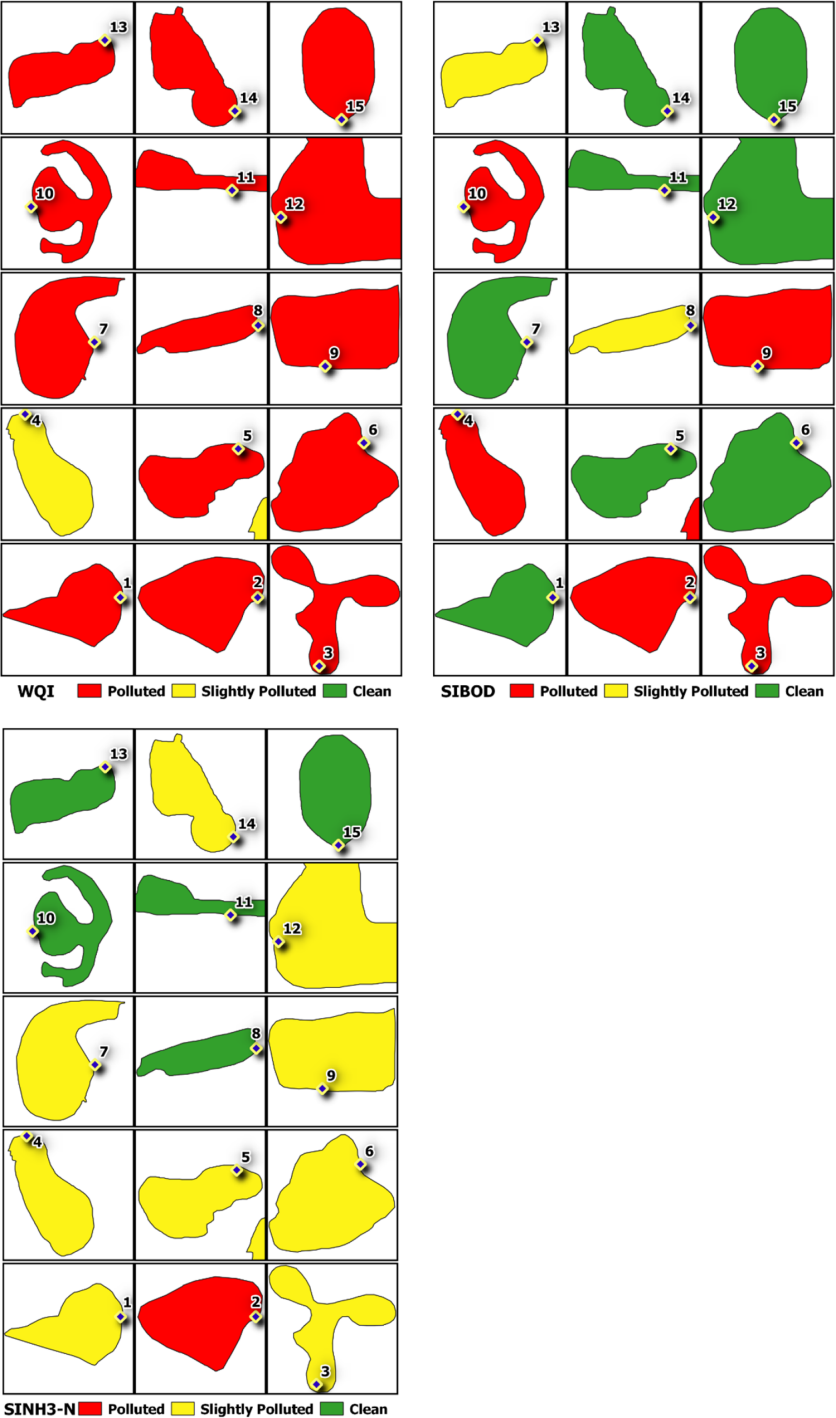
Spatial maps show the classifications of lakes based on their WQI, SIBOD and SINH3-N values

The calculated WQI ranged from 38.700 to 51.788, with lake 1 having the highest value and lake 8 having the lowest value. Based on DOE water quality classification, all 15 lakes are classified under class IV (31.0–51.9), indicating that they are suitable for irrigation use, and these values are presented in Supplementary 9. Apart from that, these lakes were also classified using DOE water quality classification based on WQI, and this is presented in Fig. 4. Fourteen lakes fall under the “polluted” index range (0–59), whereas 1 lake falls in the “slightly polluted” index (60–80). Under the same classification, the calculated sub-index (SIBOD and SIAN) used to achieve the final WQI could also be further classified into different pollution index ranges. For SIBOD, lakes 1, 5, 6, 7, 11, 12, 14, and 15 are “clean” (91–100), whereas lakes 8 and 13 are “slightly polluted” (80–90), and lakes 2, 3, 4, 8, 9, and 10 are “polluted” (0–79), where this classification is presented in Fig. 4. Meanwhile for SIAN, the “clean” (92–100) lakes are lakes 8, 10, 11, 13, and 15, while lakes 1, 3, 4, 5, 6, 7, 9, 12, and 14 are “slightly polluted” (71–91), whereas lake 2 is the only “polluted” (0–70) lake. The classification for this is presented in Fig. 4.

#### Irrigation suitability

RSC values higher than the ideal value (1.25 meq/L) would result in poor quality of soil for irrigation (Kumar et al. 2016), and from this, the two lakes having “medium” values would be less suitable for irrigation use (Table 5). High concentration of HCO_3_^−^ in waters would cause the precipitation of Ca^2+^ and Mg^2+^ to likely to occur as the water in the soil becomes more concentrated, which is shown through RSC (Ravikumar and Somashekar 2012). Only lake 8 has “poor” SAR value, indicating the adsorption of Na^+^ onto the soil cation exchange sites, causing high Na^+^ concentration, which is an unwanted feature in water used in irrigation (Aminiyan et al. 2016; Liu et al. 2012). Continuous use of water with higher SAR values would break down the physical structure of soil particles (Sappa et al. 2014). Permeability of soil is affected by the HCO_3_^−^, Ca^2+^, Mg^2+^, and Na^+^ contents in the soil, where these ions also affect the irrigation water quality on long-term use, and these are presented through PI values (Chaabane et al. 2017). Excessive amounts of Mg^2+^ would cause the water to be more alkaline, hence greatly affecting the growth of crops, and this is indicated through MH (Dash and Kalamdhad 2021). Based on this, the 5 lakes having “unsafe” MH values would be unsuitable for irrigation use. KR represents Na^+^ measured against Ca^2+^ and Mg^2+^ (Kelly 1940; Kumar et al. 2016), and based on it, only five lakes have “safe” values that are recommended for irrigation use.

**Table 5:** Water quality classification based on CHIDAM software (Chidambaram et al. 2021a, b)

Category	Grade	Samples	Category	Grade	Samples	Category	Samples
		(*n* = 15)			(*n* = 15)		(*n* = 15)
Na% Wilcox (1955)			USGS hardness			TDS classification (USSL, 1954)	
Excellent	0–20	4	Soft	< 75	11	< 200	7
Good	20–40	0	Slightly hard	75–150	2	200–500	4
Permissible	40–60	2	Moderately hard	150–300	1	500–1500	3
Doubtful	60–80	5	Very hard	> 300	1	1500–3000	0
Unsuitable	> 80	4	IBE Schoeller (1965)			Cation facies	
Na% Eaton (1950)			(Na + K) rock- > Ca/Mg g.w		1	Ca-Mg facies	4
Safe	< 60	6	(Na + K) g.w.- > Ca/Mg rock		14	Ca-Na facies	11
Unsafe	> 60	9				Na-Ca facies	0
S.A.R. Richards (1954)			Schoeller classification (1967)			Na facies	0
Excellent	0–10	12	Type I	13		Anion facies	
Good	10–18	2	Type II	0		HCO_3_ facies	0
Fair	18–26	0	Type III	2		HCO_3_-Cl-SO_4_ facies	0
Poor	> 26	1	Type IV	0		Cl-SO_4_-HCO_3_ facies	8
R.S.C Richards (1954)			Corrosivity ratio (1990)			Cl-facies	7
Good	< 1.25	13	Safe	< 1	15	Hardness classification (Handa, 1964)	
Medium	1.25–2.5	2	Unsafe	> 1	0	Permanent hardness (NCH)	
Bad	> 2.5	0	Chloride classification (Stuyfzand, 1989)			A1	0
EC Wilcox (1955)			Extremely fresh	0		A2	0
Excellent	< 250	5	Very fresh	0		A3	6
Good	250–750	5	Fresh	7		Temporary hardness (CH)	
Permissible	750–2250	3	Fresh brackish	4		B1	0
Doubtful	2250–5000	1	Brackish	3		B2	0
Unsuitable	> 5000	1	Brackish-salt	1		B3	5
			Salt	0			
			Hyperhaline	0			

**Fig. 5:**
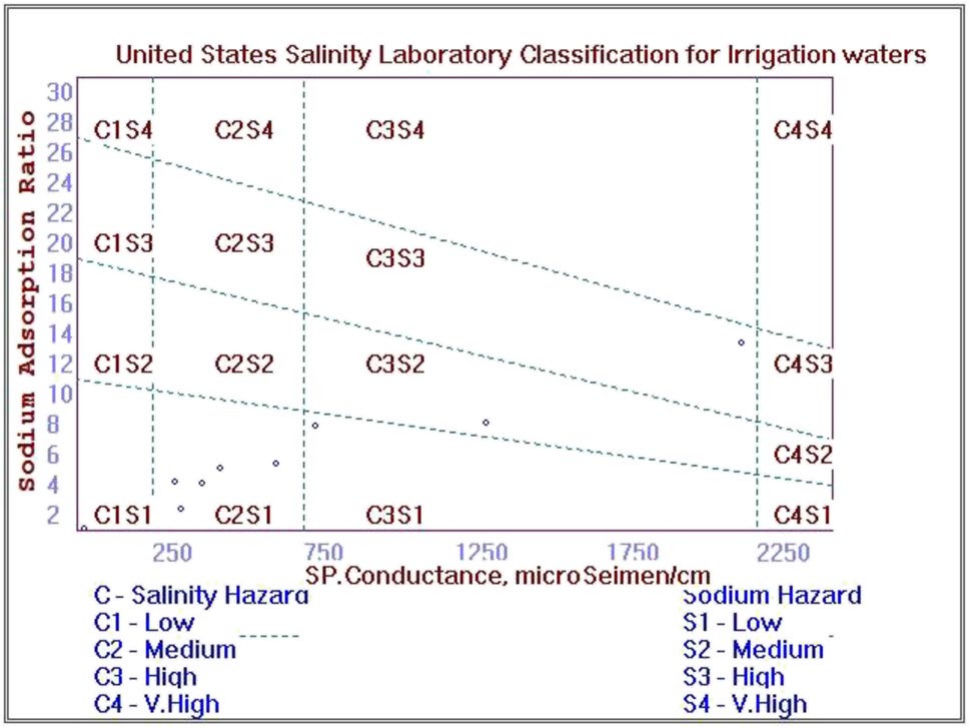
United States Salinity Laboratory (USSL) classification for irrigation water quality

**Fig. 6:**
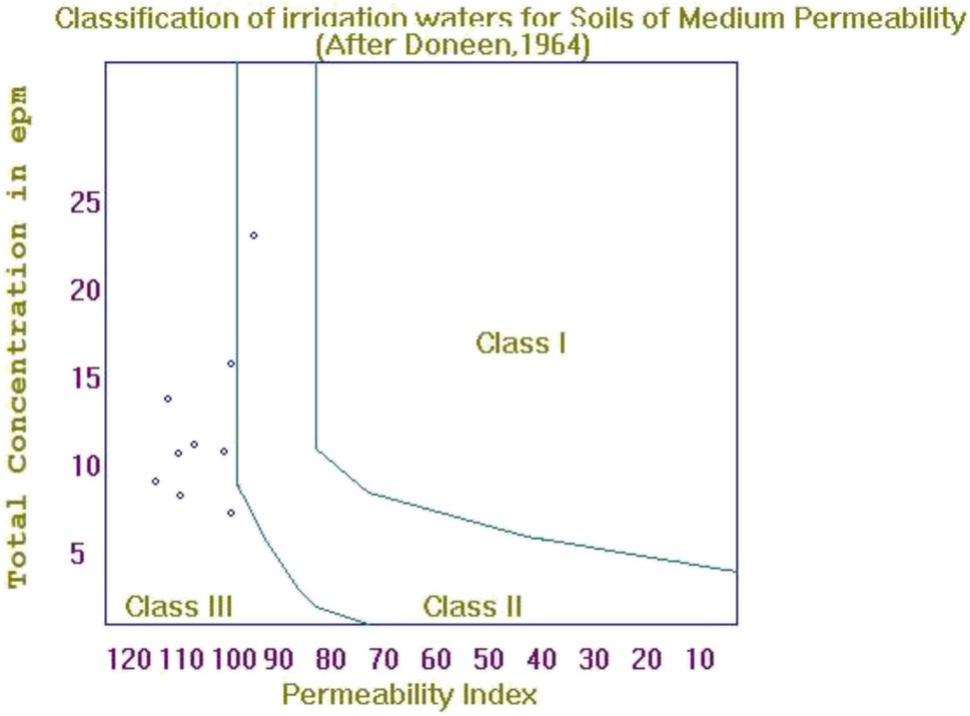
Doneen’s classification of irrigation waters based on soil permeability

In the USSL diagram (Fig. 5), the EC values are plotted against the SAR values, where the increase in EC values could be caused by Na^+^ and K^+^, instead of Ca^2+^ and Mg^2+^ (Chidambaram et al. 2021b). In the figure, 1 sample falls within the C1S1 zone (low salinity, low sodium), indicating that the water is usable for irrigation uses in most soil types. However, a higher number of the samples are within the C2S1 zone (medium salinity, low sodium). The occurrence of damage to salt-sensitive plants is caused by medium salinity hazard, and it would be necessary to do occasional flushing with low salinity water (Chaabane et al. 2017). Minor hazards might happen for low sodium water. One sample is within the C3S1 zone (high salinity, low sodium), which is still applicable for irrigation uses with the discretion of the exchangeable character of sodium (Kumar et al. 2016). With one sample within the C3S2 zone (high salinity, moderate sodium), such water type could be suitable for plants after special consideration such as maintaining good irrigation system and soil management. Water samples under high salinity classes (C3 and C4) are not recommended for irrigation use (Aminiyan et al. 2018), whereas samples under the C2S1 class are appropriate for use on all types of soil without the threat of exchangeable sodium (Sakram and Adimalla 2018). A Doneen plot is also constructed, as presented in Fig. 6. It shows that 8 samples fall within the class III field, whereas only one sample point is within the class II field. The water classes contemplated to be suitable and good for irrigation use are classes I and II, with class III considered to be unsuitable (Brindha et al. 2014; Doneen 1964).

#### Corrosivity ratio

Corrosivity ratio (CR) delivers information regarding the water supply, where water supply with a CR value lesser than 1 would be recommended and safe to be transported in any type of pipes, while water supply with a CR value greater than 1 shows that the water has a corrosive nature (Abed et al. 2021). Due to such nature, the water type would lead to corrosive effects on metal pipes. In this study, all 15 lakes record CR values greater than 1 (Table 5), making them unsafe for use, as they would corrode the insides of metal pipes. Therefore, these lakes would be unsuitable for industrial purposes due to high CR values.

### Metal pollution 

In this study, the dominance of heavy metals is as follows: Fe > Zn > Pb > Cu > Mn. As the overall concentrations of these heavy metals are considerably low, their presence in the water could be from natural sources. Based on Fig. 1 and field observation, no industrial areas were located near these lakes; hence, it could be deduced that they do not receive any industrial sewage. High Fe concentration could be the result of ferromagnetism processes and chemical weathering and dissolution (Kumar et al. 2016; Banajarani et al. 2020), which was recorded at lake 13. Naturally occurring Fe^2+^ and Mn^2+^ are found in minerals, rocks, and soils, where they are also generally soluble as they are dependent on the amount of oxygen available in water (Shamsuddin et al. 2019; Thivya et al. 2021). Mn is also said to occur together with Fe. Mn concentrations for all 15 lakes were below 0.1 mg/L, and according to Shamsuddin et al. (2019), this could be caused by the dilution and oxidation processes in the water. The Zn concentrations in all the lakes were low, which further supports that the presence of this heavy metal is not caused by anthropogenic sources. The presence of Zn in the lakes could be from the degradation of soil particles featuring Zn, aside from it occurring naturally in water (Tengku Ibrahim et al. 2020). The concentrations of Cu and Pb in all the lakes were also very low, indicating that their existence in the water is the result of natural occurrences, instead of anthropogenic activities. In Fig. 3, lake 8 has high values for both HEI and HPI, and referring to Fig. 1, the heavy metals present in this lake are from natural occurrences, as this lake is mainly controlled by the intrusion of seawater and ionic exchange processes. Lake 6 has “medium” HPI and HEI values, and similar to lake 8, the heavy metals existing in the water are caused by ionic exchange, but with a minor contribution from anthropogenic sources, as a residential area is located near the lake, as shown in Fig. 1.

### Hydrochemical processes

**Fig. 7:**
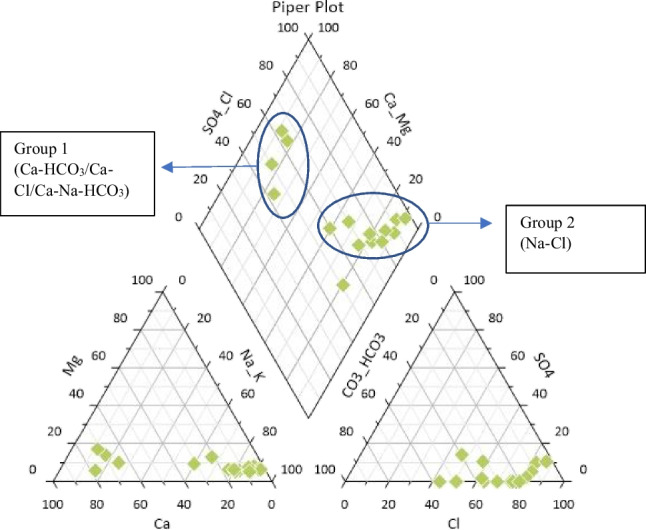
Piper plot with the classification of major water types in 15 lakes

The general chemistry of a water sample is distinguished through a Piper plot, as the plot assists in determining the similarities and differences between samples, and the evolution of water chemistry (Safari et al. 2020). Two triangles, each representing cations and anions, would later be combined to obtain a single point displayed in a diamond-shaped field, where the inference is represented on the basis of the hydrogeochemical facies concept (Mallick 2017). In the Piper plot (Fig. 7), the cation triangle shows 4 samples (lakes 1, 3, 4, 5) that are clustered within the Ca-type field, while the other 11 samples are clustered within the Na + K-type field. For anion, most of the samples are focused within the Cl-type field, whereas one sample (lake 3) is within the HCO_3_-type field. In the diamond field, the majority of the samples are clustered in the NaCl-type field, while some samples were scattered among the CaCl, CaHCO_3_, and CaNaHCO_3_ fields. Lake 5 is within the CaCl-type field, while lakes 3 and 4 are within the CaHCO_3_-type field, and lake 1 is within the CaNaHCO_3_-type field. Fourteen lakes have their strong acids (SO_4_^2−^, Cl^−^) exceeding their weak acid (HCO_3_^−^), except for lake 3. Apart from that, most lakes have their alkali (Na^+^) exceeding their alkaline earths (Ca^2+^, Mg^2+^), whereas 4 samples (lakes 1, 3, 4, 5) had their alkaline earths exceeding their alkalis. As shown in Fig. 7, the samples are divided into two groups based on their types. Samples under group 1 (lakes 1, 3, 4, 5) in the CaCl/CaHCO_3_/CaNaHCO_3_ types indicate fresh recharge water with less ionic concentration. Lakes 3 and 4 fell in the CaHCO_3_-type due to rock-water interaction or have received natural recharge from rain source (Prasanna et al. 2010; Thivya et al. 2013). Aside from that, group 2 samples (lakes 2, 6, 7, 8, 9, 10, 11, 12, 13, 14, 15) indicate high ionic strength, where they would have been derived from anthropogenic impacts and mineral dissolution (Kumar et al. 2016). This water type would also normally cause issues for drinking and irrigation purposes (Safari et al. 2020). NaCl-type is a result of higher salinities due to various anthropogenic activities (Marandi and Shand 2018). From the cation and anion dominance, it could denote that the dominant water type is NaCl-type, as it is also shown in the Piper plot, with most of the samples falling within the NaCl-type. With Na^+^ being the most dominant cation followed by Mg^2+^, this indicates that alkali metals are more predominant than alkaline earth metals (Chidambaram et al. 2021b). It is also expressed that Cl^−^ is the dominant anion, followed by HCO_3_^−^, indicating that strong acids are higher in concentration compared to weak acids.

**Fig. 8:**
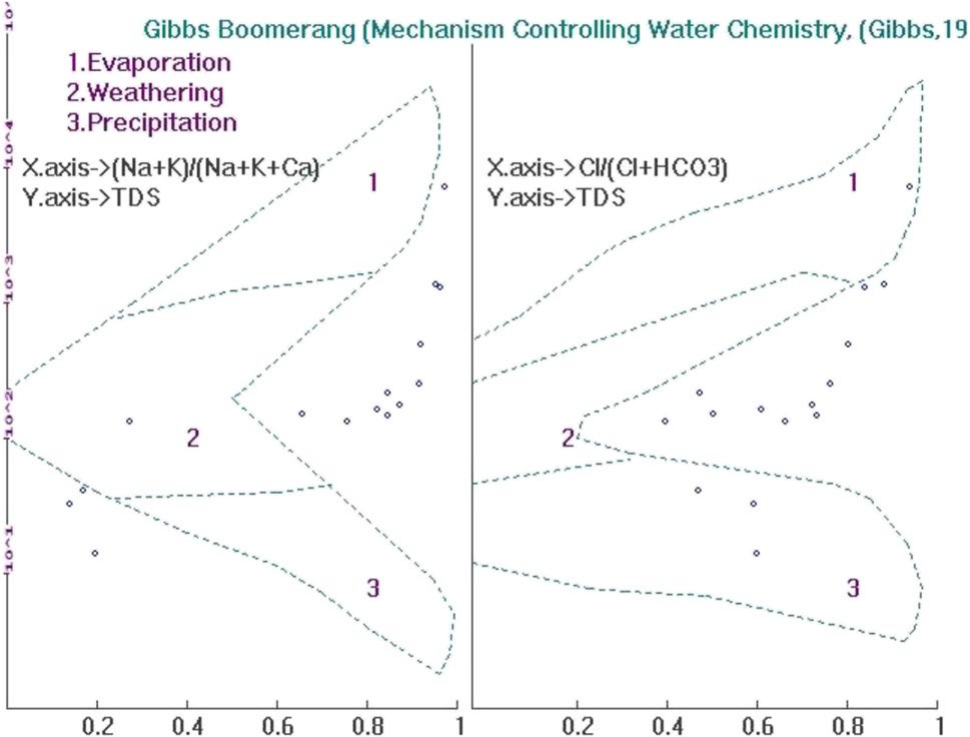
Gibbs diagram for three major processes controlling the water chemistry in 15 lakes

The source of ionic concentrations in the surface water is generally deduced using the Gibbs diagram based on three major water chemistry mechanisms according to the variation of Na/(Na + Ca), (Na + K)/(Na + K + Ca), and Cl/(Cl + HCO_3_) weight ratios as functions of TDS (Safari et al. 2020). The three mechanisms are atmospheric precipitation dominance, rock dominance, and evaporation dominance. In Fig. 8, the Gibbs diagram comprises two separate diagrams for cations and anions, respectively. Most of the samples fall in the weathering zone, indicating that the lake water chemistry is derived from the dissolution of minerals. The dominance of water–rock interaction is indicated through the samples mostly within the weathering zone (Paul et al. 2019). However, there are also samples falling outside the solid line, indicating other influencing factors, such as human influence, evaporation, and cation exchange (Singh et al. 2005; Jiang et al. 2020). These factors had a definite impact on the major ions existing in the water. From this, it suggests that the dissolution of rock through which the water is circulating is the major source of mineral components in the water (Ravikumar and Somashekar 2012; Stephan et al. 2022). Besides that, few samples have fallen either within the evaporation or precipitation zone. Samples falling within the evaporation zone could indicate that the water is affected by seawater (Shamsuddin et al. 2019).

**Fig. 9:**
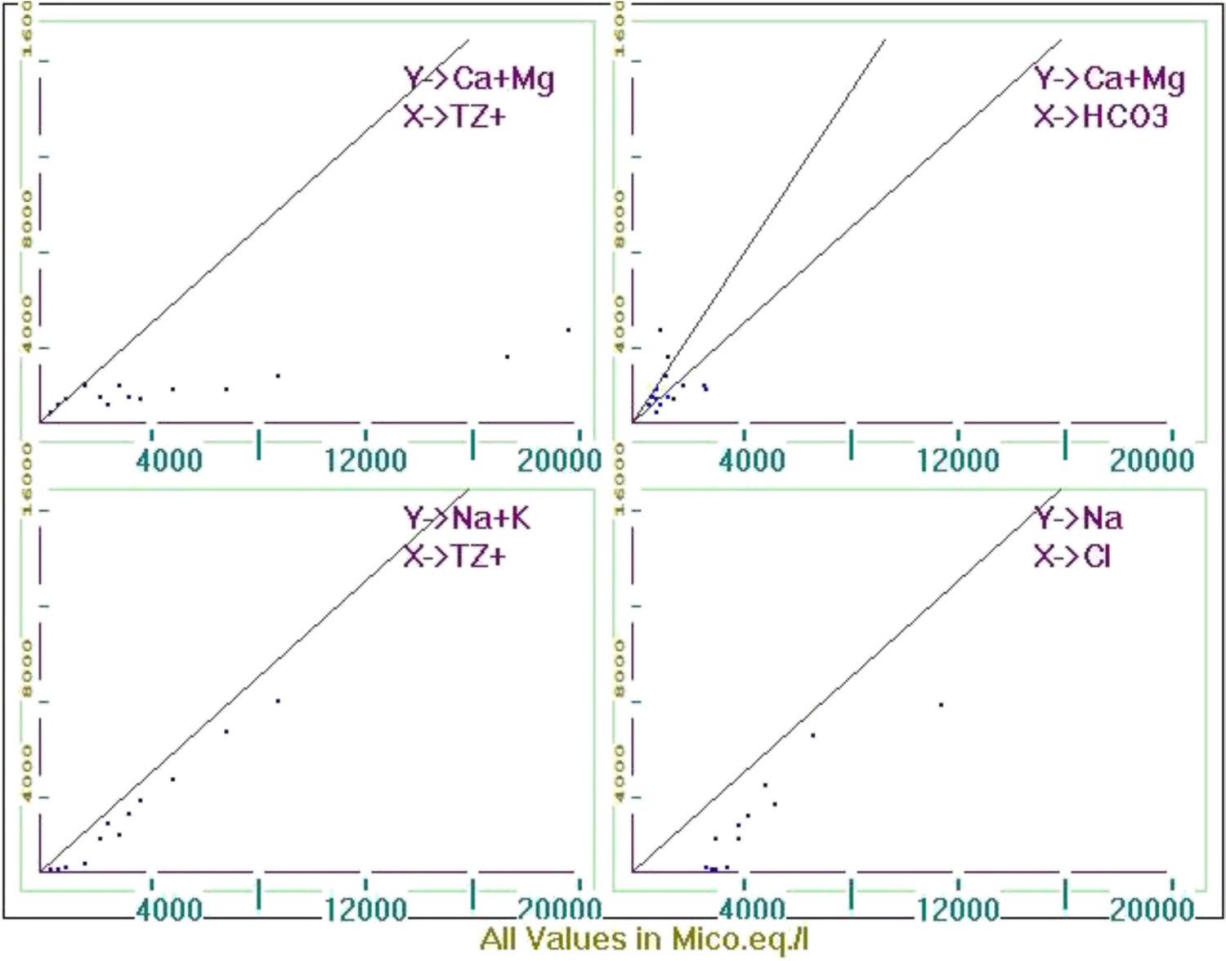
Ionic ratio plots to indicate the corresponding geochemical processes

The concentration of ions and their interrelationships represent the chemical classification (Paul et al. 2019). In Fig. 9, majority of samples fall below the equiline 1:1 in the (Ca + Mg) vs HCO_3_^−^ plot, showing the predominance of HCO_3_^−^ over alkali earth due to intense weathering of rocks (Paul et al. 2019), which agrees with the Gibb’s diagram results. Besides that, all the samples fall below the equiline 1:1 in the (Ca + Mg) vs TZ^+^ plot, indicating the dominance of total cations over alkaline earth. All of the samples in the (Na + K) vs TZ^+^ plot fall below the equiline 1:1, demonstrating the dominance of total cations over alkali earth. The Na/Cl ratio represents the dominance of Cl^−^ indicating the ion exchange in most of the samples and intrusion of saline water in lake 8 (Prasanna et al. 2010).

Ion exchange is also an important mechanism in distinguishing the surface water’s chemistry by replacing the ions in the solid face of aquifers with the ions in the solution (Safari et al. 2020). Water’s chemical properties may be significantly influenced by chemical interactions with the ionic compounds present in minerals, as well as the salinity of surface water. The dissolved Ca^2+^ and Mg^2+^ ions in the water frequently replace the cations from clay mineral ions and vice versa. Chloro-alkaline indices generally represent the ion exchange between the host environment and the source of water during transport (Abed et al. 2021). These indices were presented as CAI-I and CAI-II (Wu et al. 2015), and the equations for these indices are as below:

3 $$CAI-I=\frac{{Cl}^{-}-({Na}^{+}+{K}^{+})}{{Cl}^{-}}$$

4 $$CAI-II=\frac{{Cl}^{-}-({Na}^{+}+{K}^{+})}{{SO}_{4}^{2-}+{HCO}_{3}^{-}+{CO}_{3}^{2-}+{NO}_{3}^{-}}$$

Whenever the Ca^2+^ or Mg^2+^ ions present in the water undergo an exchange process with K^+^ or Na^+^ ions within the host rocks, the indices would achieve negative values, while positive values would be achieved when the ion exchange is reversed. As weathering was discerned as the primary controlling process in the lake water, these indices would be helpful in determining the degree of base exchange during weathering (Schoeller 1965; Paul et al. 2019). Only lake 8 showed a negative value, while the rest of the samples had positive values. From this, all 14 lakes showing positive values indicate that Ca^2+^ and Mg^2+^ ions in the rocks were exchanged for Na^+^ and K^+^ in the water, causing the increase of Ca^2+^ and Mg^2+^ concentrations in the lake water. The positive values indicate a reverse ion exchange process occurs during weathering (Paul et al. 2019).

### Multivariate analysis

The good correlation of Cl^−^ with Ca^2+^, Mg^2+^, and Na^+^ indicates the likelihood of the influence of ion exchange processes and anthropogenic activities occurring in the area (Subramani et al. 2009; Thivya et al. 2013, 2015). The good correlation of pH with SO_4_^2−^ could indicate the dissolution of sulphate minerals during the weathering process (Paul et al. 2019). The good correlation of Ca^2+^ with Mg^2+^ shows that either simple cation exchange reaction or dissolution of carbonate minerals from host rocks had occurred within the waters (Paul et al. 2019). For the 1^st^ factor, strong loadings of Na^+^ indicate the occurrence of natural weathering of minerals, along with ion exchange process in the waters (Vasanthavigar et al. 2012; Devaraj et al. 2020). Strong loadings of Ca^2+^ and Mg^2+^ indicate that silicate weathering from source rocks is the dominant process. Besides that, the 1^st^ factor has high loading of EC, Ca^2+^, Mg^2+^, Na^+^, K^+^, Cl^−^, and SO_4_^2−^, indicating that highly dissolved concentrations of these major ions have direct influence on high EC values (Khadija et al. 2021). The 2^nd^ factor included high positive loadings of NO_3_^−^ and NH_3_-N, which could be related to anthropogenic sources such as domestic sewage (Khadija et al. 2021).

**Fig. 10:**
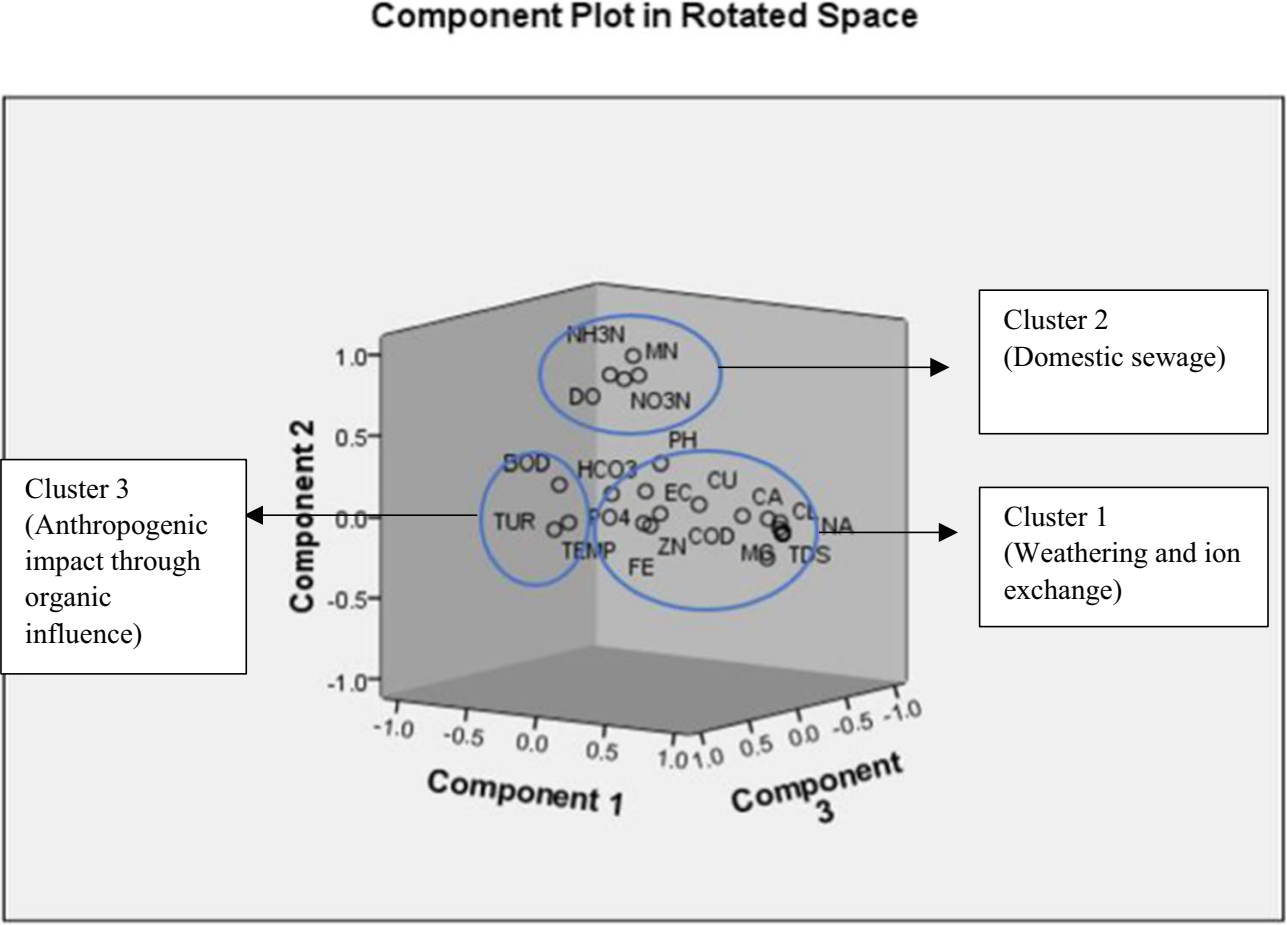
Major geochemical processes in the lake waters

**Fig. 11:**
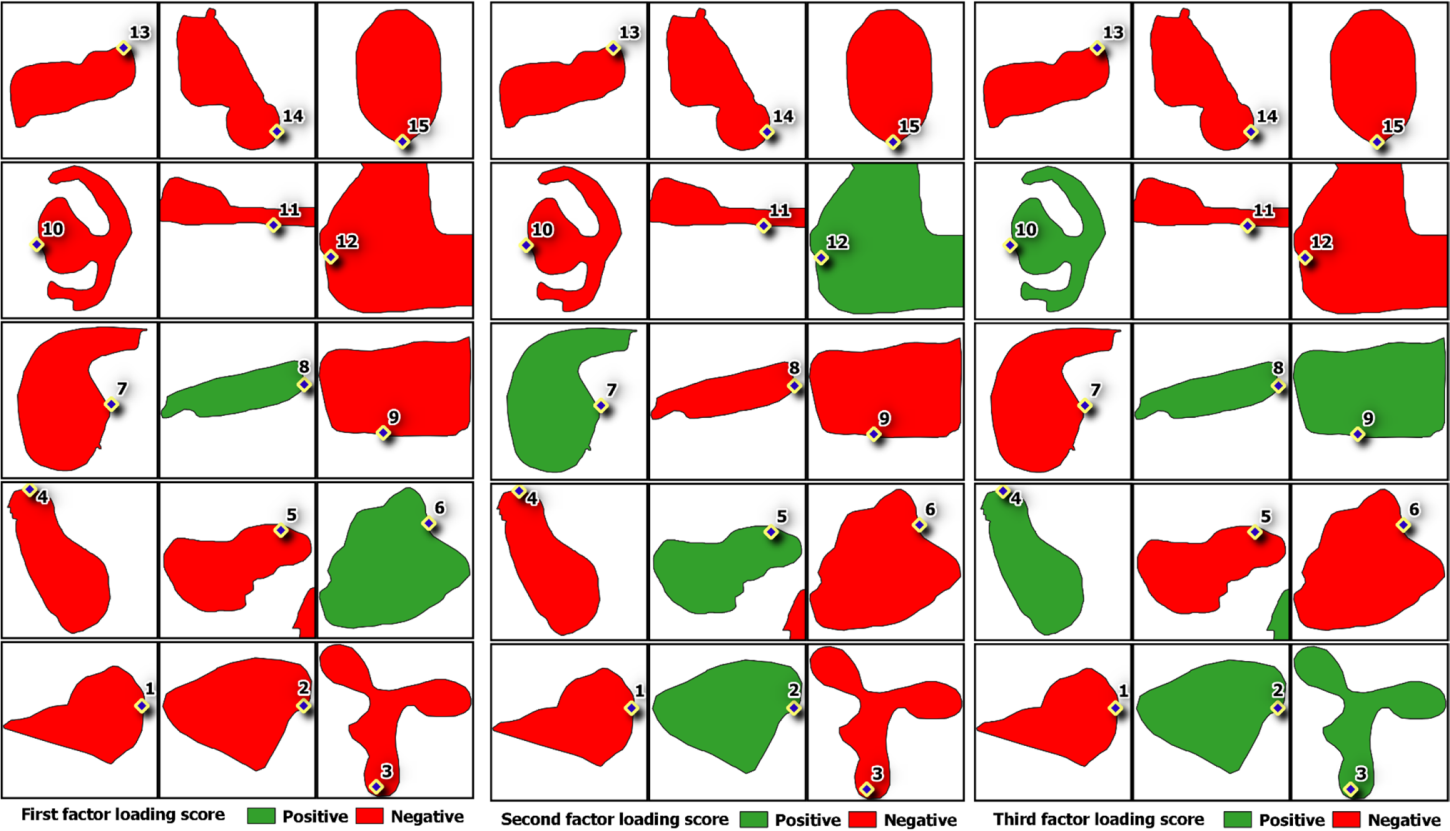
Factor scores for the 15 lakes

In Fig. 10, the plots could be divided into three clusters based on the key processes that control the lake water quality. The 1^st^ cluster is primarily composed of major ions, heavy metals, pH, EC, TDS, PO_4_^3−^, and COD, while the 2^nd^ cluster contains DO, NO_3_^−^, NH_3_-N, and Mn, and the 3^rd^ cluster comprises of turbidity, temperature, and BOD. The 1^st^ cluster is predominantly affected by natural processes, such as the intrusion of saline water, ionic exchange, and dissolution of minerals, as this cluster consists of major ions and heavy metals, along with EC and TDS. The higher values of these parameters were recorded at lake 8, which receives seawater through the inlet to the lake. Anthropogenic activities could also have contributed to this cluster with influence. The source assumed to contribute to the 2^nd^ cluster is majorly from anthropogenic sources such as domestic sewage, especially with this cluster having both NO_3_^−^ and NH_3_-N (Khadija et al. 2021). For the 3^rd^ cluster, anthropogenic activities with organic influence play a role for this cluster, as it includes BOD and turbidity. The highest concentrations of these two parameters were recorded at lake 10, where this lake receives sewage from the food stalls nearby. High BOD and turbidity values were recorded at the lakes receiving domestic sewage and other human activities. In addition, the six factor scores were evaluated to remark their impacts on the 15 lakes (Fig. 11), where positive values were reviewed as having impacts on the lakes. Factor 1 showed impact on lakes 6 and 8, while factor 2 influenced lakes 2, 5, 7, and 12. Lakes 2, 3, 4, 8, 9, and 10 were affected by factor 3, whereas factor 4 influenced lakes 2, 3, 4, 8, 9, and 13. Besides that, factor 5 had affected lakes 6, 7, 10, 11, and 15, while factor 6 brought effects to lakes 6, 8, 10, 12, 13, and 15.

## Conclusion

The water quality assessment of the 15 lakes within Miri City has shown that the dominance of major ions is as follows: for anion Cl^−^  > HCO_3_^−^  > SO_4_^2−^, while for cation Na^+^  > Mg^2+^  > K^+^  > Ca^2+^. From this, it further confirms the water types of these lakes, with the majority of the lakes being the NaCl-type, while a few lakes are scattered within the CaCl, CaHCO_3_, and CaNaHCO_3_ fields. The main controlling mechanisms of these waters are weathering, ionic exchange processes, and anthropogenic impacts, including saline water intrusion in lake 8. The nutrients in the lakes are resulted from anthropogenic activities such as domestic sewage, whereas low concentrations of heavy metals present could be due to natural occurrences and less likely by anthropogenic activities. In terms of irrigation use, lakes 1, 2, 3, 4, and 5 would be preferably suitable based on SAR, Na%, RSC, PI, KR, and MH. As for the level of heavy metal pollution, lakes 1, 2, 3, 4, 5, and 15 are considered safe, as both HPI and HEI values at these lakes have classified them as “low” degree of pollution. As for industrial use, none of the lakes is suitable as their CR values have exceeded the safe value, hence would cause the inside of metal pipes to corrode. According to NWQS, all 15 lakes are classified as suitable for irrigation use (class IV). Based on their WQI values, 14 lakes fall under the “polluted” index, with 1 lake under the “slightly polluted” index. From the multivariate analysis, it is inferred that the lake water quality is affected by both geogenic and anthropogenic impacts. Based on the outcome of this study, it is concluded that few lakes are in alarming status, thus deemed unsuitable for the Miri community. Hence, a proper management plan is needed to safeguard this water resource.

### Supplementary Information

Below is the link to the electronic supplementary material.

Supplementary file1 (DOCX 36 KB)

## Data Availability

The datasets used and analysed during the current study are available from the corresponding author on reasonable request.
