# Supplementary material for: Assessment on urban lakes along the coastal region of Miri, NW Borneo: implication for hydrochemistry, water quality, and pollution risk
Source: Environ Sci Pollut Res Int. 2023 Jan 18;31(29):41306–28. doi: 10.1007/s11356-023-25172-9 (PMC11219409; doi:10.1007/s11356-023-25172-9)
Supplement: Supplementary file 1 — Supplementary file1 (DOCX 36 KB) [file 11356_2023_25172_MOESM1_ESM.docx]

**Supplementary 1**. List of lakes visited, with their location names, coordinates and brief description of the lakes

| Lake no. | Location name | Coordinates | Brief description |
| --- | --- | --- | --- |
| 1 | Kpg. Lusut | 4°18’48.2” N  114°0,33.55” E | Lake water having clear appearance, as water is see-through. Presence of fishes and aquatic plants in the water. No water flow in/out, no other water bodies connected to the lake or located nearby.  Dimension: 130 × 195 = 25, 350 m^2^ |
| 2 | Jln. Airport | 4°20’16.98” N  113°59’6.14” E | Lake water having dirty, green coloured appearance. Not receiving any water flow from other water bodies, and no other water bodies located nearby. May have presence of aquatic life within the water.  Dimension: 60 × 80 = 4800 m^2^ |
| 3 | Jln. Kipas | 4°24’9.65” N  113°59’39.98” E | Lake water had a dirty appearance. Obvious signs of fishes living in the waters. Lake not connected to any other water bodies, and no other water bodies were observed nearby.  Dimension: 60 × 80 = 4800 m^2^ |
| 4 | Tmn. Bulatan Lake (a) | 4°25’12.04” N  114°0’51.16” E | Lake water did not have clear appearance, as colour of water was olive green. Presence of aquatic life in the water. Not connected to Lake 5 despite being at the same location. Water appeared still/motionless.  Dimension: 100 × 100 = 10, 000 m^2^ |
| 5 | Tmn. Bulatan Lake (b) | 4°25’12.97” N  114°0’50.15” E | Lake water had a dirty appearance, with murky green coloured water. Presence of aquatic life in the water. No water flow in/out, and water was still and motionless.  Dimension: 84 × 84 = 7056 m^2^ |
| 6 | Go-Kart Lake | 4°26’44.30” N  114°2’52.80” E | Lake water had dark-brownish colour, with plants and dead leaves floating on the surface. Water flow only occurred within the lake and assumed no water flow in/out of the lake, making the lake appearing still/motionless. No other water bodies seen nearby.  Dimension: 690 × 690 = 476, 100 m^2^ |
| 7 | South Lake | 4°26’28.97” N  114°2’13.78” E | Lake water did not have a clear appearance, with the water having dark green to brownish colour. Water flow only occurs within the lake, and not connected to other water bodies. The lake is located within a residential area, but not near any houses.  Dimension: 540 × 360 = 194, 400 m^2^ |
| 8 | Jln. Pantai | 4°26’54.92” N  114°0’0.22” E | Lake water had dirty and murky appearance, with rubbish and turbidity floating on the water surface. Water was observed to be motionless/still. The lake receives seawater from a small inlet from the sea. The lake is located opposite a residency area, separated by a road.  Dimension: 200 × 50 = 10, 000 m^2^ |
| 9 | Jln. Maigold | 4°29’23.53” N  114°0’51.73” E | Lake water had a dirty appearance and was green-dark green colour. Rubbish were floating on the surface, especially near land. No other water bodies were found nearby, and the lake was not connected to any other water bodies. The water seem still, and minor wave motions occurred with the lake. Lake located opposite a neighbourhood.  Dimension: 270 × 180 = 48, 600 m^2^ |
| 10 | Kolam MGP | 4°29’51.07” N  114°0’40.72” E | Lake water having dirty appearance and was dark green. No water flow in/out of the lake, as not connected to other water bodies. The lake receives sewage from the food stalls located within the location area.  Dimension: 180 × 270 = 48, 600 m^2^ |
| 11 | Jln. Curtin (a) | 4°30’20.05” N  114°1’5.38” E | Lake was not see-through, as the water was dark brown to black. Presence of fishes in the water and plants floating on the surface. Water seem to be still, and only flow within the lake do occur. Lake could be connected to Lake 12 but separated by a road and wide area of land.  Dimension: 1440 × 360 = 518, 400 m^2^ |
| 12 | Jln, Curtin (b) | 4°30’22.82” N  114°1’22.33” E | Lake water had a dirty appearance and the water having dark brown to black colour. Rubbish was found flowing on the surface, especially near land. Presence of aquatic life. The lake is located near a residency area.  Dimension: 237 × 237 = 56, 169 m^2^ |
| 13 | Within Curtin | 4°30’39.28” N  114°1’13.22” E | Lake water is brown in colour and had a dirty appearance. Aquatic life present in the water. The lake is not connected to other water bodies, making the lake isolated.  Dimension: 160 × 80 = 12, 800 m^2^ |
| 14 | Curtin main lake | 4°30’52.81” N  114°1’0.23” E | Lake did not have a clear appearance, but instead quite dirty and could not see through the surface. Lake had a colour of dark brown-black. Aquatic life present, and plants found floating on the surface.  Dimension: 1200 × 600 = 720, 000 m^2^ |
| 15 | Curtin Lakeside | 4°30’42.26” N  114°0’55.01” E | Lake water was not clean, as some rubbish was found floating on the surface. Water having a dirty appearance, was dark brown-black colour, and could not see through the surface. The lake receives water flow from Lake 14, as these 2 lakes are connected. Small pipes conveying drainage/sewage into the lake.  Dimension: 60 × 100 = 6000 m^2^ |

**Supplementary 2**. Irrigation indices used in this study, along with their respective equations.

| Indices | Equation |
| --- | --- |
| Sodium adsorption ratio (SAR) | $SAR=\frac{{Na}^{+}}{\sqrt{\frac{{(Ca}^{2+}+{Mg}^{2+})}{2}}}$ |
| Residual sodium carbonate (RSC) | $RSC=\left[ {HCO}_{3}^{-}+{CO}_{3}^{2-} \right]-[{Ca}^{2+}+{Mg}^{2+}]$ |
| Percentage sodium (%Na) | $\%Na=\frac{({Na}^{+}+K^{+})}{{Ca}^{2+}+{Mg}^{2+}+{Na}^{+}K^{+}}\times100\%$ |
| Magnesium hazard (MH) | $MH=\frac{{Mg}^{2+}}{{Ca}^{2+}+{Mg}^{2+}}\times100$ |
| Permeability index (PI) | $PI=\frac{({Na}^{+}+\sqrt{{HCO}_{3}^{-})}}{{Ca}^{2+}+{Mg}^{2+}+{Na}^{+}}\times100$ |
| Kelly’s ratio (KR) | $KR=\frac{{Na}^{+}}{{Ca}^{2+}+{Mg}^{2+}}$ |

**Supplementary 3**. Pollution indices related to heavy metal, used in this study

| Pollution indices | Equation |
| --- | --- |
| Heavy metal pollution index (HPI) | $HPI=\frac{\sum_{i=1}^{n} W_{i}Q_{i}}{\sum_{i-1}^{n} W_{i}}$  The unit weights and sub-indices for the chosen metals are represented by the W_i_ and Q_i_, and these are calculated by the equations below:  $W_{i}=\frac{K}{S_{i}}=\frac{1}{S_{i}}$  $Q_{i}=\sum_{i=1}^{n} \frac{(M_{i}-I_{i})}{(S_{i}-I_{i})}\times100$  The M_i_, I_i_, and S_i_ would represent the monitored value of metal, ideal and standard values of the ith parameter, respectively, and the (-) symbol indicate the numerical difference between the two numbers, but the algebraic sign is usually ignored. |
| Heavy metal evaluation index (HEI) | $HEI=\sum_{i=1}^{n} \frac{H_{c}}{H_{max}}$  The H_c_ represents the concentration of a heavy metal, while the H_max_ represents the maximum allowed concentration of each metal of the ith sample. |

**Supplementary 4.** Calculated values of irrigation indices for the 15 lakes.

| Sample | Irrigation indices | | | | | |
| --- | --- | --- | --- | --- | --- | --- |
|  | SAR | Na% | RSC | PI | KR | MH |
| 1 | 0.112 | 12.8 | 0.602 | 234 | 0.125 | 24.8 |
| 2 | 1.5 | 50.4 | 0.508 | 97.2 | 0.866 | 33.1 |
| 3 | 0.147 | 16.1 | 1.25 | 110 | 0.0848 | 19.8 |
| 4 | 0.138 | 12 | 0.703 | 137 | 0.103 | 11 |
| 5 | 0.108 | 8.95 | 0.403 | 146 | 0.0914 | 28.3 |
| 6 | 11.3 | 81 | -2.66 | 85.9 | 4.13 | 76.1 |
| 7 | 12.5 | 84.7 | -1.27 | 91.3 | 5.41 | 77.6 |
| 8 | 38 | 86.1 | -18.2 | 86.7 | 6.01 | 82.5 |
| 9 | 3.18 | 70.5 | 0.406 | 106 | 2.26 | 39.7 |
| 10 | 4.18 | 76.7 | 0.106 | 103 | 3.12 | 44.2 |
| 11 | 7.1 | 79 | -0.583 | 91.5 | 3.66 | 62.9 |
| 12 | 1.84 | 57.3 | -0.193 | 95.7 | 1.31 | 49.7 |
| 13 | 4.43 | 74.5 | 1.51 | 108 | 2.75 | 38.2 |
| 14 | 3.2 | 73.5 | 0.00452 | 105 | 2.72 | 42.6 |
| 15 | 6.98 | 81.7 | -0.29 | 95.8 | 4.34 | 53.6 |

**Supplementary 5**. Classification of water, based on irrigation indices.

| Sample | Irrigation indices | | | | | | |
| --- | --- | --- | --- | --- | --- | --- | --- |
|  | SAR | Na% | Na% | RSC | PI | KR | MH |
| 1 | Excellent | Excellent | Safe | Good | Class I | Safe | Safe |
| 2 | Excellent | Permissible | Safe | Good | Class I | Safe | Safe |
| 3 | Excellent | Excellent | Safe | Medium | Class I | Safe | Safe |
| 4 | Excellent | Excellent | Safe | Good | Class I | Safe | Safe |
| 5 | Excellent | Excellent | Safe | Good | Class I | Safe | Safe |
| 6 | Good | Unsuitable | Unsafe | Good | Class I | Unsafe | Unsafe |
| 7 | Good | Unsuitable | Unsafe | Good | Class I | Unsafe | Unsafe |
| 8 | Poor | Unsuitable | Unsafe | Good | Class I | Unsafe | Unsafe |
| 9 | Excellent | Excellent | Unsafe | Good | Class I | Unsafe | Safe |
| 10 | Excellent | Excellent | Unsafe | Good | Class I | Unsafe | Safe |
| 11 | Excellent | Excellent | Unsafe | Good | Class I | Unsafe | Unsafe |
| 12 | Excellent | Permissible | Safe | Good | Class I | Unsafe | Safe |
| 13 | Excellent | Excellent | Unsafe | Medium | Class I | Unsafe | Safe |
| 14 | Excellent | Excellent | Unsafe | Good | Class I | Unsafe | Safe |
| 15 | Excellent | Unsuitable | Unsafe | Good | Class I | Unsafe | Unsafe |

**Supplementary 6**. Calculated HPI and HEI values, the maximum, minimum and average values, along with their respective level of degree of pollution (DOP) of the 15 lake waters.

| Sample | Location name | HPI | DOP | HEI | DOP |
| --- | --- | --- | --- | --- | --- |
| 1 | Kpg. Lusut | 42.6 | Low | 1.3 | Low |
| 2 | Jln. Airport | 3.6 | Low | 0.8 | Low |
| 3 | Jln. Kipas | 5.2 | Low | 0.6 | Low |
| 4 | Tmn Bulatan Lake (a) | 22.6 | Low | 0.7 | Low |
| 5 | Tmn Bulatan Lake (b) | 12.7 | Low | 0.6 | Low |
| 6 | Go-Kart Lake | 91.9 | Medium | 2.1 | Medium |
| 7 | South Lake | 77.6 | Medium | 1.3 | Low |
| 8 | Jln. Pantai | 284.8 | High | 4.1 | High |
| 9 | Jln. Maigold | 68.1 | Medium | 1.3 | Low |
| 10 | Kolam MGP | 61.7 | Medium | 1.7 | Low |
| 11 | Jln. Curtin (a) | 72.4 | Medium | 1.7 | Low |
| 12 | Jln. Curtin (b) | 42.7 | Low | 2.0 | Medium |
| 13 | Within Curtin | 45.5 | Low | 4.2 | High |
| 14 | Curtin main lake | 20.7 | Low | 2.0 | Medium |
| 15 | Curtin Lakeside | 36.7 | Low | 1.8 | Low |
| Max. |  | 284.8 |  | 4.2 |  |
| Min. |  | 3.6 |  | 0.6 |  |
| Avg. |  | 59.3 |  | 1.7 |  |

**Supplementary 7**. Correlation analysis

**Supplementary 8.** Classification of lakes according to the National Lake Water Quality Criteria and Standards.

| Sample | Location | Category |
| --- | --- | --- |
| 1 | Kpg. Lusut | D |
| 2 | Jln. Airport | C, D |
| 3 | Jln. Kipas | C, D |
| 4 | Tmn. Bulatan Lake (a) | B, C, D |
| 5 | Tmn. Bulatan Lake (b) | A, B, C, D |
| 6 | Go-Kart Lake | D |
| 7 | South Lake | D |
| 8 | Jln. Pantai | D |
| 9 | Jln. Maigold | C, D |
| 10 | Kolam MGP | B, C, D |
| 11 | Jln. Curtin (a) | B, C, D |
| 12 | Jln. Curtin (b) | A, B, C, D |
| 13 | Within Curtin | A, B, C, D |
| 14 | Curtin main lake | D |
| 15 | Curtin Lakeside | B, C, D |

**Supplementary 9**. Calculated values of WQI for the 15 lakes.

| Sample | SIDO | SIBOD | SICOD | SIAN | SipH | WQI |
| --- | --- | --- | --- | --- | --- | --- |
| 1 | 0 | 98.7503 | 61.0429 | 79.5 | 94.446955 | 51.78806 |
| 2 | 100 | 67.16104 | 42.69886 | 2.781845 | 71.001856 | 50.52992 |
| 3 | 0 | 67.71824 | 59.03637 | 78.45 | 99.488917 | 46.01846 |
| 4 | 100 | 63.87749 | 31.52002 | 79.5 | 71.001856 | 59.62515 |
| 5 | 0 | 98.9195 | 13.58985 | 86.85 | 90.855395 | 44.89923 |
| 6 | 0 | 94.6049 | 13.269 | 88.95 | 97.7548 | 45.17105 |
| 7 | 0 | 92.363 | 33.29517 | 86.85 | 99.600397 | 47.85574 |
| 8 | 0 | 84.8336 | 11.43337 | 91.05 | 59.126784 | 38.70044 |
| 9 | 0 | 73.06603 | 43.44807 | 82.65 | 88.703875 | 43.8762 |
| 10 | 0 | 60.98896 | 39.81079 | 99.45 | 87.709195 | 43.40023 |
| 11 | 0 | 98.0735 | 12.95251 | 96.3 | 96.486325 | 46.72973 |
| 12 | 0 | 99.0041 | 8.686355 | 90 | 98.553472 | 45.52701 |
| 13 | 0 | 85.4258 | 24.66389 | 99.45 | 98.930653 | 46.9663 |
| 14 | 0 | 99.8924 | 23.25641 | 75.3 | 98.110925 | 45.76889 |
| 15 | 0 | 99.5963 | 24.66389 | 97.35 | 99.072292 | 49.36069 |
